# Implementation of Health IT for Cancer Screening in US Primary Care: Scoping Review

**DOI:** 10.2196/49002

**Published:** 2024-04-30

**Authors:** Constance Owens-Jasey, Jinying Chen, Ran Xu, Heather Angier, Amy G Huebschmann, Mayuko Ito Fukunaga, Krisda H Chaiyachati, Katharine A Rendle, Kim Robien, Lisa DiMartino, Daniel J Amante, Jamie M Faro, Maura M Kepper, Alex T Ramsey, Eric Bressman, Rachel Gold

**Affiliations:** 1 BRIDGE-C2 Implementation Science Center in Cancer Control Oregon Health & Science University Portland, OR United States; 2 Department of Health Administration and Policy College of Public Health George Mason University Fairfax, VA United States; 3 OCHIN, Inc Portland, OR United States; 4 Department of Preventive Medicine and Epidemiology Chobanian & Avedisian School of Medicine Boston University Boston, MA United States; 5 Data Science Core, Chobanian & Avedisian School of Medicine Boston University Boston, MA United States; 6 iDAPT Implementation Science Center for Cancer Control Wake Forest School of Medicine Winston Salem, NC United States; 7 Department of Medicine Massachusetts General Hospital Boston, MA United States; 8 Department of Family Medicine Oregon Health & Science University Portland, OR United States; 9 Adult and Child Center for Outcomes Research and Delivery Science, Ludeman Family Center for Women’s Health Research Division of General Internal Medicine University of Colorado School of Medicine Aurora, CO United States; 10 Department of Medicine UMass Chan Medical School Worcester, MA United States; 11 Penn Implementation Science Center in Cancer Control Perelman School of Medicine University of Pennsylvania Philadelphia, PA United States; 12 Verily Life Sciences South San Francisco, CA United States; 13 Leonard Davis Institute of Health Economics University of Pennsylvania Philadelphia, PA United States; 14 Milken Institute School of Public Health George Washington University Washington, DC United States; 15 RTI International Research Triangle Park, NC United States; 16 UT Southwestern Medical Center University of Texas Dallas, TX United States; 17 Department of Population and Quantitative Health Sciences UMass Chan Medical School Worcester, MA United States; 18 Brown School Washington University St. Louis, MO United States; 19 Department of Psychiatry Washington University School of Medicine St. Louis, MO United States; 20 Perelman School of Medicine University of Pennsylvania Philadelphia, PA United States; 21 Kaiser Permanente Center for Health Research Portland, OR United States

**Keywords:** cancer prevention, health information technology, implementation, implementation strategies, scoping review

## Abstract

**Background:**

A substantial percentage of the US population is not up to date on guideline-recommended cancer screenings. Identifying interventions that effectively improve screening rates would enhance the delivery of such screening. Interventions involving health IT (HIT) show promise, but much remains unknown about how HIT is optimized to support cancer screening in primary care.

**Objective:**

This scoping review aims to identify (1) HIT-based interventions that effectively support guideline concordance in breast, cervical, and colorectal cancer screening provision and follow-up in the primary care setting and (2) barriers or facilitators to the implementation of effective HIT in this setting.

**Methods:**

Following scoping review guidelines, we searched MEDLINE, CINAHL Plus, Web of Science, and IEEE Xplore databases for US-based studies from 2015 to 2021 that featured HIT targeting breast, colorectal, and cervical cancer screening in primary care. Studies were dual screened using a review criteria checklist. Data extraction was guided by the following implementation science frameworks: the Reach, Effectiveness, Adoption, Implementation, and Maintenance framework; the Expert Recommendations for Implementing Change taxonomy; and implementation strategy reporting domains. It was also guided by the Integrated Technology Implementation Model that incorporates theories of both implementation science and technology adoption. Reporting was guided by PRISMA-ScR (Preferred Reporting Items for Systematic Reviews and Meta-Analyses extension for Scoping Reviews).

**Results:**

A total of 101 studies met the inclusion criteria. Most studies (85/101, 84.2%) involved electronic health record–based HIT interventions. The most common HIT function was clinical decision support, primarily used for panel management or at the point of care. Most studies related to HIT targeting colorectal cancer screening (83/101, 82.2%), followed by studies related to breast cancer screening (28/101, 27.7%), and cervical cancer screening (19/101, 18.8%). Improvements in cancer screening were associated with HIT-based interventions in most studies (36/54, 67% of colorectal cancer–relevant studies; 9/14, 64% of breast cancer–relevant studies; and 7/10, 70% of cervical cancer–relevant studies). Most studies (79/101, 78.2%) reported on the reach of certain interventions, while 17.8% (18/101) of the included studies reported on the adoption or maintenance. Reported barriers and facilitators to HIT adoption primarily related to inner context factors of primary care settings (eg, staffing and organizational policies that support or hinder HIT adoption). Implementation strategies for HIT adoption were reported in 23.8% (24/101) of the included studies.

**Conclusions:**

There are substantial evidence gaps regarding the effectiveness of HIT-based interventions, especially those targeting guideline-concordant breast and colorectal cancer screening in primary care. Even less is known about how to enhance the adoption of technologies that have been proven effective in supporting breast, colorectal, or cervical cancer screening. Research is needed to ensure that the potential benefits of effective HIT-based interventions equitably reach diverse primary care populations.

## Introduction

### Background

For common cancer types such as cervical, colorectal, and breast cancer, routine screening provided in primary care settings can save lives [1]. Although evidence-based national guidelines exist for the provision of such screenings [1-4], patient receipt of guideline-concordant cancer screening is suboptimal nationally and varies substantially across clinical settings [5,6]. This is driven by multiple factors, including provider-level barriers such as the challenge of staying current on changing cancer screening guidelines [6] and the cognitive overload that providers can face when managing the needs of patients with complex conditions [7-11]. Patient-level barriers include lack of knowledge of screening recommendations [6], loss to follow-up [12], fear about screening procedures or outcomes, and financial and logistical challenges [13].

Understanding which interventions effectively address these challenges—and the barriers and facilitators to implementing such interventions—is needed to enhance the delivery of guideline-concordant cancer screening in primary care. The Community Preventive Services Task Force summary of evidence-based interventions for addressing barriers to guideline-concordant cancer screening [14] identifies health IT (HIT)–based interventions as showing particular promise [15-17]. Prior systematic reviews found that HIT-based interventions such as patient reminders and provider feedback tools can be effective in supporting cancer prevention care [15,17,18]. Such interventions can enhance provider-patient communication about cancer screening [19-22]. These interventions can also help care teams identify patients due for screening with automated reminders embedded in the electronic health record (EHR) that can appear either at the point of care [23] and during panel or population management [24].

Yet HIT-based interventions targeting numerous health outcomes are underused in primary care settings [23,25]. One recent systematic review involving 55 studies showed that clinical decision support tools were adopted in <35% of eligible encounters [26]. The adoption of such interventions is impeded by multilevel barriers, such as the challenges inherent to integrating new tools into clinical workflows [27], and lack of training in how to use such tools [28,29]. There is a need to understand best practices for enhancing the adoption of effective HIT-based interventions targeting cancer prevention, including how barriers to the adoption of such interventions can best be addressed in primary care [17,18,30,31].

### Objectives

In 2020, the National Cancer Institute’s Consortium for Cancer Implementation Science (CCIS) “Technology in Implementation Science Action Group” identified a need for the scoping review presented here. This review aims to describe the specific knowledge gaps in this evidence base, that is, what is known and unknown about the implementation of effective HIT for cancer screening in primary care. Specifically, it aims to identify (1) HIT-based interventions that effectively support guideline concordance in breast, cervical, and colorectal cancer screening provision and follow-up in the primary care setting and (2) barriers or facilitators to the implementation of effective HIT in this setting. To refine the scope of this review, we focused on common cancer screenings that are in the purview of primary care: breast, colorectal, and cervical cancer screening. We note that earlier systematic reviews [15,17,18] assessed the effectiveness of HIT-based interventions at improving cancer screening rates in primary care, but the most recent included data up to June 2014 [15]. This review first summarizes related evidence accrued since 2014 and then assesses current knowledge on the adoption of such interventions. To our knowledge, this is the first scoping review to assess the implementation of HIT in cancer screening.

## Methods

### Overview

This scoping review was conducted by a multidisciplinary team of researchers from the CCIS with expertise in implementation science, health informatics, health services research, and cancer control. We followed the 6-stage scoping review methodology described by Arksey and O’Malley [32], with consideration of later modifications to this approach made by Levac et al [33]. This review was reported in accordance with the PRISMA-ScR (Preferred Reporting Items for Systematic Reviews and Meta-Analyses extension for Scoping Reviews) [34].

### Ethical Considerations

Ethics approval from the George Mason University Institutional Review Board was not required for this review.

### Research Questions

This scoping review was designed to answer two overarching questions: (1) What is known about how HIT-based interventions are used to enhance guideline concordance of cancer screening in primary care settings? (2) What is known about the barriers or facilitators to the implementation and dissemination of these interventions?

### Identifying Relevant Studies

With assistance from a health sciences librarian, the first author (COJ) conducted a 3-step search process to identify relevant US-based peer-reviewed and gray literature studies. First, the following bibliographic databases were systematically searched: MEDLINE, CINAHL Plus, Web of Science, and IEEE Xplore. These databases were searched using a combination of search strings that included relevant controlled vocabulary (eg, Medical Subject Heading) and keywords with Boolean operators. The search terms were selected based on a review of the existing literature and refined based on the input of the coauthors. To ensure that the search yielded relevant studies, variations of the search strategy were pilot-tested by 3 authors (COJ, RG, and RX) and refined before the final search was conducted. Our final search strategy for bibliographic databases is provided in Multimedia Appendix 1.

Second, this search was supplemented by a review of gray literature (eg, study protocols, unpublished empirical trials, dissertations, reports, and government publications) to consider studies that might not be indexed in bibliographic databases. This search primarily consisted of targeted website searching of cancer, HIT, public health organizations, and funding agencies recommended by the authors (COJ, RG, KHC). Our final gray literature search strategy is provided in Multimedia Appendix 2. Additional gray literature databases (CQ Press Library, Policy File Index, Find Policy, and Harvard Kennedy School Think Tank Search), recommended by the health sciences librarian, were explored but did not yield useful results. Finally, we identified relevant studies with a snowball search technique, whereby the reference lists of sources selected for full-text review were also examined for additional studies to include in the final review sample.

### Study Selection

#### Eligibility Criteria

Studies on HIT and cancer screening before January 2015 are covered in prior publications [15,17,18]. Our search was designed to build on that work, so it was limited to studies published from 2015 to 2021 (the time at which we started the review process). Studies were considered eligible for inclusion if they (1) were US-based, reported in the English language, and published between January 2015 and June 2021; (2) reported on activities conducted in the primary care setting; (3) focused on evidence-based cancer screening; (4) involved the use of HIT to support this screening; (5) were related to specific workflow steps involved in conducting cancer screening in primary care (identifying patients due for screening at the point of care or in panel management, obtaining results of past screenings through data exchange, or providing appropriate follow-up care); and (6) targeted screening for breast, colorectal, or cervical cancer. A checklist of these criteria was created to guide the selection of relevant studies and then pilot-tested in a subsample of articles (n=60) and refined (COJ, RG, and RX) to ensure that its criteria could be applied consistently. The checklist was supported by a glossary of key terms to ensure shared understanding across reviewers of potential studies. The final checklist and glossary are provided in Multimedia Appendices 3 and 4, respectively. All study designs were eligible for inclusion as long as the study included some description of how HIT was used to support breast, colorectal, and cervical cancer screening in primary care settings. If a study was an evidence review (eg, systematic review or narrative review), only studies included in the final sample of the review and published between January 2015 and June 2021 were assessed for potential inclusion. If multiple publications described a single intervention but described different approaches for using HIT, all applicable studies were assessed for inclusion.

#### Dual Screening Review

Results of the search strategies described above were imported and managed in Zotero [35]. The first author (COJ) removed duplicate studies. Then, reviewers in eight 2-person teams were assigned studies to dual screen [36] (team 1: COJ and RG; team 2: AH and HA; team 3: LD and RX; team 4: KR and JMF; team 5: KHC and EB; team 6: KAR and JC; team 7: MMK and ATR; and team 8: MIF and DJA). Dual screening was performed in 2 steps. First, study titles and abstracts were dual screened by each review team using the inclusion and exclusion checklist to assess eligibility. Second, studies included for full-text dual screening were assessed by the same review teams for final inclusion in the scoping review. Any discrepancies that emerged within a review team were reconciled by consensus. The first and senior authors (COJ and RG) provided final decisions for any studies that could not be reconciled by a review team.

### Data Charting

A data charting form was developed using Qualtrics, a web-based survey software, to systematically extract information from studies selected for inclusion in the review analyses. The form was initially pilot-tested on 2 articles and refined (COJ, RG, and HA). Next, the review teams extracted information from their assigned studies. Extracted data elements included study citation, publication year, publication type, study design, study setting, sample composition by race or ethnicity, and cancer screening focus (breast, colorectal, and cervical cancer). Extracted characteristics of the relevant HIT tools involved in a given study included type, users, functions, purpose (intervention or implementation strategy supporting an intervention), and supported cancer screening activities. Data elements were extracted in multiple choice or free-text form, depending on the type of data. Multiple implementation frameworks [37-40] were used to guide data extraction. A check of at least 50% (49/101 studies) of extracted studies suggested that data charting quality was high and the agreement rate between the initial reviewers and the reviewers that conducted the quality check was >90%.

Multiple implementation frameworks [37-40] were used to guide data extraction. Specifically, the Reach, Effectiveness, Adoption, Implementation, and Maintenance (RE-AIM) framework [37] guided the extraction of dissemination and implementation outcomes: target end users (clinical staff and patients) of HIT (Reach), HIT impact on cancer screening in primary care (Effectiveness), the rate of HIT adoption (Adoption), the extent to which a given HIT-based intervention was implemented (Implementation), and the extent to which sustainability of HIT adoption was measured (Maintenance). Assessment of the evidence on barriers and facilitators of HIT adoption was guided by the Integrated Technology Implementation Model (ITIM), which includes 12 inner and outer context concepts known to be central to the implementation and adoption of technology in health care settings, and is based on the Consolidated Framework for Implementation Research, adapted to HIT-based interventions [38]. Although technology frameworks have been used to investigate the usability and acceptance of HIT-based interventions [41-43], to our knowledge, the ITIM is the only model that incorporates theories of both implementation science and technology adoption. The Expert Recommendations for Implementing Change (ERIC) compilation [39] guided the categorization of discrete implementation strategies identified in the studies. The implementation strategies reporting the framework by Proctor et al [40] guided the extraction and analysis of implementation strategies used to support the HIT adoption.

### Collating, Summarizing, and Reporting Results

Descriptive data were compiled and interpreted using Stata/MP (version 15.1; StataCorp LLC) to quantify the frequencies of extracted data in discrete fields. Free text data charted in Qualtrics were exported to Excel (Microsoft Corp) for qualitative content analysis [44,45]. Authors (COJ, JC, RX, and RG) reviewed and categorized free text for HIT characteristics, RE-AIM domains, implementation barriers, facilitators, and core elements of implementation strategies (eg, actor and target of action). Most analyses used an iterative process, which involved initial coding and identification of themes (ie, categories) by 2 reviewers, resolving discrepancies and refining categories through team discussion, and recoding the text using finalized categories. Multimedia Appendix 5 provides details about these procedures.

### Consultation

Authors (RG, JC, RX, HA, and AH) were consulted at each stage of the scoping review to provide input on the search, data abstraction, and interpretation of the results. We also consulted with implementation science experts about the conceptual frameworks selected for this study.

## Results

### Literature Search

**Figure 1:**
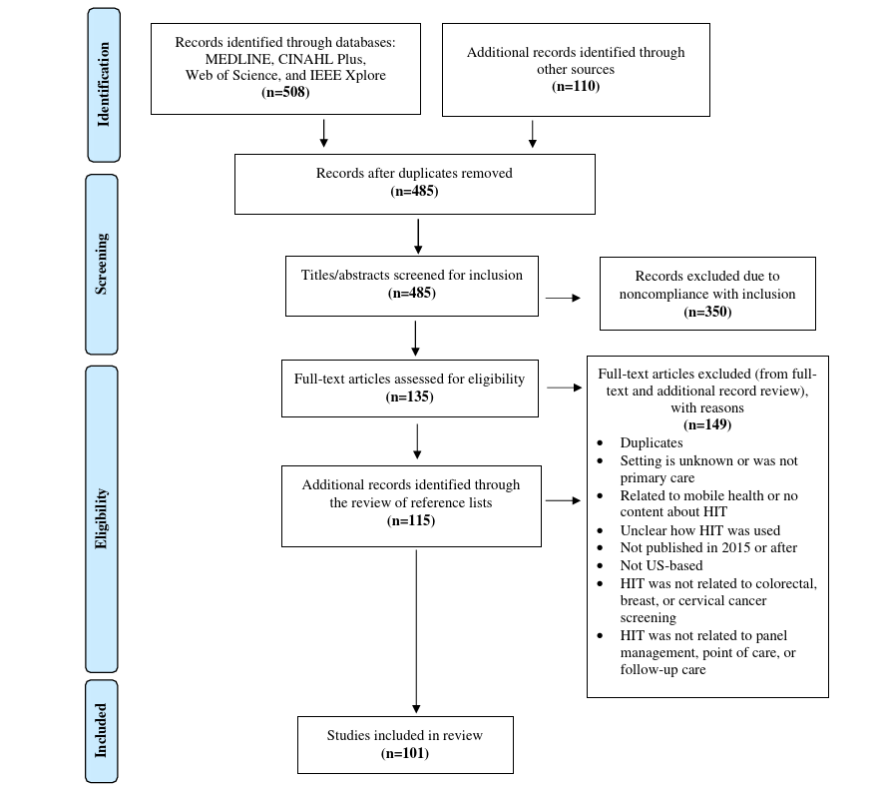
PRISMA (Preferred Reporting Items for Systematic Reviews and Meta-Analyses) flow diagram. HIT: health IT.

The search yielded an initial total of 618 studies (Figure 1). After removing duplicates, 485 titles and abstracts were assessed for inclusion. Among these, 350 studies were excluded as not meeting the inclusion criteria. Full-text review was conducted on 135 records that met the inclusion criteria. A snowball search yielded an additional 115 studies that were assessed for eligibility. A final total of 101 studies met the inclusion criteria. Multimedia Appendix 6 provides a complete list of these studies.

### Characteristics of the Included Studies

Included studies were published between January 2015 and June 2021 (Table 1). Most studies were peer-reviewed (92/101, 91.1%). Study design was mostly nonexperimental (descriptive: 18/101, 17.8% or observational: 15/101, 14.9%) in comparison to experimental (randomized controlled trials: 29/101, 28.7%), quasi-experimental (pre-post design: 21/101, 20.8%; nonrandomized controlled trials: 5/101, 5%; or other quasi-experimental studies: 3/101, 3%), and other studies (10/101, 9.9%). Most studies covered HIT targeting colorectal cancer screening (83/101, 82.2%), followed by breast cancer screening (28/101, 27.7%) and cervical cancer screening (19/101, 18.8%); these sum up >101 as some addressed more >1 type of cancer screening.

**Table 1:** Characteristics of the included studies (N=101).

Characteristics			Colorectal cancer (n=83), n (%)^a^	Breast cancer (n=28), n (%)	Cervical cancer (n=19), n (%)	Total, (N=101), n (%)
**Publication year^b^**						
	2015		13 (15.7)	4 (14.3)	1 (5.3)	15 (14.9)
	2016		15 (18.1)	6 (21.4)	3 (15.8)	18 (17.8)
	2017		15 (18.1)	5 (17.9)	4 (21.1)	21 (20.8)
	2018		16 (19.3)	2 (7.1)	3 (15.8)	18 (17.8)
	2019		10 (12)	5 (17.9)	4 (21.1)	13 (12.9)
	2020		9 (10.8)	5 (17.9)	3 (15.8)	11 (10.9)
	2021		5 (6)	1 (3.6)	1 (5.3)	5 (5)
**Publication type**						
	Peer-reviewed article		78 (94)	26 (92.9)	16 (84.2)	92 (91.1)
	Report		1 (1.2)	2 (7.1)	2 (10.5)	4 (4)
	Study protocol		3 (3.6)	—^c^	—	3 (3)
	Other		1 (1.2)	—	1 (5.3)	2 (2)
**Study design**						
	**Nonexperimental**					
		Descriptive	15 (18.1)	5 (17.9)	4 (21.1)	18 (17.8)
		Observational	11 (13.3)	9 (32.1)	4 (21.1)	15 (14.9)
	**Experimental**					
		RCT^d^	24 (28.9)	7 (25)	5 (26.3)	29 (28.7)
	**Quasi-experimental**					
		Pre-post study design	17 (20.5)	5 (17.9)	2 (10.5)	21 (20.8)
		Non-RCT	3 (3.6)	1 (3.6)	3 (15.8)	5 (5)
		Other quasi-experimental	3 (3.6)	—	—	3 (3.0)
	Other study designs		10 (12)	1 (3.6)	1 (5.3)	10 (9.9)

^a^Percentages were calculated based on column totals.

^b^Publication year represents studies published from January 2015 to June 2021.

^c^Not available.

^d^RCT: randomized controlled trial.

Characteristics of the primary care settings where the research in the included studies was conducted are shown in Table 2. Approximately half of the included studies (52/101, 51.5%) reported on practice location. Most studies involving colorectal (22/83, 27%) or breast (6/28, 21%) cancer screening were conducted in urban areas, and most studies on cervical cancer screening (5/19, 26%) were conducted in rural areas. Studies on colorectal cancer screening were primarily conducted in federally qualified health centers (20/83, 24%); most of those on breast and cervical cancer screening were conducted in academic-based clinics (9/28, 32% and 5/19, 26%, respectively). More than half (59/101, 58.4%) of the included studies (colorectal: 47/83, 57%; breast cancer: 17/28, 61%; and cervical: 8/19, 42%) reported information on racial or ethnic minoritized participants (patients from racial or ethnic minority groups). Of these 59 studies, 34 (58%) reported that ≤50% of study participants were members of racial or ethnic minority populations.

**Table 2:** Primary care practice characteristics of the included studies (N=101).

Characteristics		Colorectal cancer (n=83), n (%)^a^	Breast cancer (n=28), n (%)	Cervical cancer (n=19), n (%)	Total, (N=101), n (%)
**Practice location**					
	Urban	22 (26.5)	6 (21.4)	2 (10.5)	26 (25.7)
	Rural	11 (13.3)	5 (17.9)	5 (26.3)	15 (14.9)
	Combination of urban and rural	11 (13.3)	2 (7.1)	3 (15.8)	11 (10.9)
	Not reported	39 (47)	15 (53.6)	9 (47.4)	49 (48.5)
**Practice type**					
	Academic-based clinic	17 (20.5)	9 (32.1)	5 (26.3)	22 (21.8)
	Federally Qualified Health Centers	20 (24.1)	1 (3.6)	3 (15.8)	21 (20.8)
	Freestanding or other	18 (21.7)	4 (14.3)	3 (15.8)	21 (20.8)
	Hospital-based clinic	10 (12)	2 (7.1)	1 (5.3)	12 (11.9)
	Not reported	18 (21.7)	12 (42.9)	7 (36.8)	25 (24.8)
**Sample percentage of racial or ethnic minority groups**					
	≤50%	25 (30.1)	13 (46.4)	6 (31.6)	34 (33.7)
	>50%	22 (26.5)	4 (14.3)	2 (10.5)	25 (24.8)
	Not reported	36 (43.4)	11 (39.3)	11 (57.9)	42 (41.6)

^a^Percentages were calculated based on column totals.

### Characteristics of the HIT Interventions

Our definitions of HIT tool types and functions and the types of cancer screening activities they supported are provided in Multimedia Appendix 4. Of the 101 included studies, 66 (65.3%) reported on interventions involving 1 HIT tool and 35 (34.7%) reported on interventions involving >1 HIT tool (Table 3). In these studies, the HIT tool was either the intervention of focus, one component of a multicomponent intervention that also included non-HIT elements, or was used as an implementation strategy to support the intervention of focus.

Most of the included studies (85/101, 84.2%) involved EHR-based HIT tools (Table 3). Web-based (18/101, 17.8%) and other types of HIT tools (19/101, 18.8%) were less common. The HIT *function* most commonly involved in included studies was clinical decision support (CDS) across all cancer screening types (Table 3). CDS tools for panel management were most common in studies involving colorectal cancer screening (50/83, 60%). CDS at the point of care was commonly used in studies on breast (16/28, 57%) and cervical cancer screening (12/19, 63%). Other commonly studied HIT functions included risk identification (colorectal: 13/83, 16% and cervical: 6/19, 32%), patient decision aids (colorectal: 13/83, 16% and breast: 9/28, 32%), and tools for tracking patient adherence to recommended care (colorectal: 27/83, 33% and cervical: 6/19, 32%).

The *cancer screening activities* were primarily related to identifying patients for screening in panel management (colorectal: 50/83, 60%; breast: 8/28, 29%; and cervical: 7/19, 37%) and at the point of care (colorectal: 39/83, 47%; breast: 15/28, 54%; and cervical: 12/19, 63%). Other commonly supported cancer screening activities included follow-up care for referral (colorectal: 36/83, 43%; breast: 7/28, 25%; and cervical: 7/19, 37%) and for positive or abnormal screening results (colorectal: 12/83, 15% and cervical: 5/19, 26%; Table 3).

**Table 3:** Characteristics of the health IT (HIT) sources and functions used to promote cancer screening in primary care, as represented in the included studies (N=101).

Characteristics			Colorectal cancer (n=83), n (%)^a^		Breast cancer (n=28), n (%)		Cervical cancer (n=19), n (%)		Total (N=101), n (%)
**Using single or multiple HIT tools**									
	Single HIT tools	53 (63.9)		22 (78.6)		14 (73.7)		66 (65.3)	
	Multiple HIT tools	30 (36.1)		6 (21.4)		5 (26.3)		35 (34.7)	
**HIT sources**									
	EHR^b^ based	74 (89.2)		20 (71.4)		18 (94.7)		85 (84.2)	
	Web based	11 (13.3)		9 (32.1)		3 (15.8)		18 (17.8)	
	Other or unclear	15 (18.1)		3 (10.7)		3 (15.8)		19 (18.8)	
**HIT functions**									
	CDS^c^ panel management or outreach	50 (60.2)		7 (25)		9 (47.4)		57 (56.4)	
	CDS point of care	41 (49.4)		16 (57.1)		12 (63.2)		48 (47.5)	
	Risk identification	13 (15.7)		5 (17.9)		6 (31.6)		18 (17.8)	
	Patient decision aid	13 (15.7)		9 (32.1)		2 (10.5)		18 (17.8)	
	Provider assessment and feedback	11 (13.3)		1 (3.6)		1 (5.3)		12 (11.9)	
	Tracking patient adherence	27 (32.5)		4 (14.3)		6 (31.6)		30 (29.7)	
	Other	3 (3.6)		—^d^		—		3 (3.0)	
**Cancer screening activities supported by HIT**									
	Panel management	50 (60.2)		8 (28.9)		7 (36.8)		56 (55.4)	
	Point of care	39 (47)		15 (53.6)		12 (63.2)		45 (44.6)	
	Follow-up (referral)	36 (43.4)		7 (25.0)		7 (36.8)		41 (40.6)	
	Follow-up (abnormal or positive result)	12 (14.5)		2 (7.1)		5 (26.3)		17 (16.8)	
	Acquire previous results	7 (8.4)		2 (7.1)		4 (21.1)		10 (9.9)	
	Other	21 (25.3)		11 (39.3)		5 (26.3)		24 (23.8)	

^a^Percentages were calculated based on column totals. Some studies featured >1 HIT source, function, and cancer screening activity. As a result, these categories are not mutually exclusive and will not necessarily sum to 100%. Refer to Multimedia Appendix 4 for definitions of the terms used in this table.

^b^EHR: electronic health record.

^c^CDS: clinical decision support.

^d^Not available.

### Reporting on RE-AIM Outcomes

#### Overview

A summary of reporting on RE-AIM outcomes is provided in Table 4.

**Table 4:** Reporting on Reach, Effectiveness, Adoption, Implementation, and Maintenance (RE-AIM) outcomes for health IT (HIT) targeting cancer screening in primary care.

RE-AIM domains	Data charted	Cancer screening type		
		Colorectal cancer	Breast cancer	Cervical cancer
Reach	Was the number of targeted staff or patients for HIT-based intervention reported	High^a^	High	High
Effectiveness	Did the HIT tools show positive results in the cancer screening intervention	High	Moderate	High
Adoption	Rate of HIT adoption	Low	Moderate	Low
Implementation	Barriers, facilitators, and implementation strategies used related to HIT	Moderate	Low	Low
Maintenance	Was the sustainment of HIT adoption measured	Low	Low	Low

^a^Low: <25% of the included studies for each cancer screening type category, moderate: 25% to 50% of the included studies for each cancer screening type category, and high: >50% of the included studies for each cancer screening type category. Percentages were calculated with respect to the included studies for each cancer screening type category.

#### Effectiveness

Of the 101 included studies, 24 (23.8%) reported on the effectiveness of HIT targeting breast (14/28, 50% of breast cancer–relevant studies) and cervical cancer screening (10/19, 53% of cervical cancer–relevant studies; Multimedia Appendix 7 includes a table with these results). Of the 101 included studies, 54 (53.5%) reported the effectiveness of HIT targeting colorectal cancer screening (54/83, 65% of colorectal cancer–relevant studies). Among studies reporting on effectiveness, most-reported positive outcomes (improved screening rate) associated with the use of HIT (36/54, 67% of colorectal cancer–relevant studies; 9/14, 64% of breast cancer–relevant studies; and 7/10, 70% of cervical cancer–relevant studies). This evidence mostly represented CDS used during panel management (22/83, 27% of colorectal cancer–relevant studies) or at the point of care (5/28, 18% of breast cancer–relevant studies and 5/19, 26% of cervical cancer–relevant studies; Multimedia Appendix 7).

#### Reach, Adoption, and Maintenance

Among the 101 included studies, 79 (78.2%) reported on the reach of HIT-based interventions. Most of the studies focused on reach involved HIT for colorectal cancer screening (63/83, 76% of colorectal cancer–relevant studies studies). The reach of HIT-based interventions targeting breast cancer screening was reported in 82% (23/28) of the breast cancer–relevant studies and in 74% (14/19) of the studies targeting cervical cancer screening. Overall, 15.8% (16/101) of the studies reported on HIT adoption (colorectal: 10/83, 12%; breast: 9/28, 32%; and cervical: 1/19, 5%), and 2% (2/101) of the studies reported on maintenance of HIT-based interventions. Of those that reported on adoption, there was mostly a low rate of adoption (≤50%) across all cancer screening types (Multimedia Appendix 8 includes a table with these results).

#### Implementation

The proportion of studies reporting on the implementation of the HIT ranged from 25% to 50% for those related to colorectal cancer screening (Table 4). It was reported in <25% of the studies related to HIT targeting breast and cervical cancer screening. Implementation barriers, facilitators, and strategies related to HIT adoption across all cancer screening types are described further in the next 2 sections.

### Implementation Barriers and Facilitators of HIT Adoption

A total of 34 studies reported on barriers and 37 studies reported on facilitators to implementing the HIT-based interventions of focus in primary care (Table 5). The most-reported barriers and facilitators were related to the ITIM constructs inner context (barriers: 17/34, 50% and facilitators: 14/37, 38%), nature of the innovation (barriers: 15/34, 44% and facilitators: 17/37, 46%), and outer context (barriers: 11/34, 32% and facilitators: 9/37, 24%). Inner context barriers included limited staff time to use the HIT and adoption competing with other clinic priorities. Inner context facilitators included having dedicated staff assigned to operate and manage a given HIT tool, and organizational policies supporting HIT adoption. Barriers related to the nature of the innovation included inaccurate cancer screening data reported by the HIT intervention and the burden of HIT development and maintenance. Facilitators related to the nature of the innovation included that HIT automation and customization features reduced staff resources and time needed in providing care. Outer context barriers included challenges involved with working with an EHR vendor to activate and update the tool and challenges with accessing screening results conducted outside the clinics. Outer context facilitators included Medicaid expansion including cancer screening as an incentivized metric and the clinic being a Federally Qualified Health Center, which necessitated responsiveness to such metrics. A table with more examples of barriers and facilitators is provided in Multimedia Appendix 9.

**Table 5:** Reporting on the barriers and facilitators of health IT adoption aligned with the Integrated Technology Implementation Model (ITIM).

ITIM constructs	Barriers (n=34), n (%)^a^	Facilitators (n=37), n (%)
Adoption or adopters	2 (6)	1 (3)
Communication	6 (18)	5 (14)
Economic environment	5 (15)	6 (16)
Facilitators (boundary spanner)	—^b^	4 (11)
Implementation	3 (9)	9 (24)
Inner context	17 (50)	14 (38)
Interfacing systems	5 (15)	2 (5)
Leadership	2 (6)	2 (5)
Nature of the innovation	15 (44)	17 (46)
Outer context	11 (32)	9 (24)
Users (adopters)	9 (26)	4 (11)
Workflow	9 (26)	11 (30)

^a^Percentages were calculated with respect to the total studies that reported barriers or facilitators. Some studies featured both barriers and facilitators to health IT adoption for cancer screening in primary care. As a result, these categories are not mutually exclusive and will not necessarily sum to 100%.

^b^Not available.

### Implementation Strategies to Support HIT Adoption

Implementation strategies targeting HIT adoption were reported in 24% (24/101) of the included studies. Those reported were mapped to 22 implementation strategies from the ERIC compilation [39] (Multimedia Appendix 10). Of the studies reporting implementation strategies, >50% used ≥2 strategies and >50% reported strategies promoting HIT use for colorectal cancer screening. Common strategies to promote HIT use among all cancer screening types included central technical assistance, conducting small tests of change, and educational meetings. A table with more examples is available in Multimedia Appendix 10. Reported evidence mapped to the domains formulated by Proctor et al [40] (Table 6) and were mostly focused on describing implementation strategies to support HIT adoption for colorectal cancer screening (22/83, 27% of colorectal cancer–relevant studies) in comparison to breast (6/28, 21% of breast cancer–relevant studies) and cervical cancer screening (4/19, 21% of cervical cancer–relevant studies). Overall, less than half of the included studies, for each cancer screening type, reported evidence in accordance with each implementation strategy domain.

**Table 6:** Reporting on the implementation strategies used to support health IT adoption.

Implementation strategy domains by Proctor et al [40]	Data charted	Cancer screening type		
		Colorectal cancer	Breast cancer	Cervical cancer
Actor	Who delivers the strategy	Moderate^a^	Low	Low
Action	Procedures to conduct the strategy	Moderate	Low	Low
Target of action	Intent of action	Low	Low	Low
Temporality	When does the strategy happen	Low	Low	Low
Dose	Frequency or intensity	Low	Low	Low
Implementation outcomes affected	What will the strategy change	Low	Low	Low
Justification	Purpose of the strategy	Moderate	Low	Low

^a^Low: <25% of the included studies for each cancer screening type category, moderate: 25% to 50% of the included studies for each cancer screening type category, and high: >50% of the included studies for each cancer screening type category.

## Discussion

### Principal Findings

This scoping review summarizes the state of the science about the implementation of HIT-based interventions targeting breast, cervical, or colorectal cancer screening in primary care. Previous reviews identified the positive impact of HIT-based interventions throughout the cancer care continuum, including cancer screening [15,17,18]. This review adds to prior evidence by bringing an implementation science perspective; this is needed because the impact of HIT-based interventions is limited by the extent to which such interventions are effectively integrated into practice. This scoping review provides updated evidence up to 2021. This is not a systematic review; our goal was to identify knowledge gaps. Results indicate that key knowledge gaps related to the implementation of HIT in cancer screening in primary care include (1) the effectiveness of HIT targeting *breast and cervical* cancer screening, (2) HIT *adoption* in diverse primary care settings, (3) the *implementation strategies* that support the adoption of HIT, and (4) equitable reach or adoption of HIT. Addressing these evidence gaps may be critical to supporting the implementation of high-quality primary care [46].

### Knowledge Gap 1: Limited Evidence on the Effectiveness of HIT Targeting Breast and Cervical Cancer Screening

This review emphasizes the need to improve the evidence on HIT effectiveness, especially HIT targeting breast and cervical cancer screening uptake, in diverse primary care settings. Effectiveness outcomes included, but were not limited to, improvements in cancer screening initiation by the patient or provider and patient completion of cancer screening. Although the use of HIT-based interventions was associated with improved screening outcomes for all 3 cancer types, there were far fewer studies of HIT effectiveness for breast and cervical cancer screening (a combined total of 24 studies) in comparison to the 54 studies involving colorectal cancer screening. Furthermore, most studies related to HIT targeting breast or cervical cancer prevention were conducted in academic medical centers and were not readily generalizable to other primary care settings. This limited evidence is concerning as both are common cancers, and evidence-based guidelines for such screenings are not met in many patient populations.

In addition, the lack of reporting on HIT effectiveness was especially common in studies in which HIT was part of a multicomponent intervention [14]; thus, even if the effectiveness of the overall intervention was reported, the impact of the HIT element of the intervention was not clear. More research is needed to establish the effectiveness of HIT targeting cancer screening in diverse primary care settings, including trials of the individual and combined effect of HIT within multicomponent interventions. The need for an improved understanding of the effectiveness of HIT is especially salient given that national programs (eg, Promoting Interoperability Program, formerly Meaningful Use) promote the use of HIT in health care settings [47] as a means to improve health outcomes.

### Knowledge Gap 2: Limited Evidence of the Reach, Adoption, and Maintenance of Effective HIT Targeting Cancer Screening

The limited reporting on the reach, adoption, and maintenance of such interventions aligns with the known lack of reporting on these implementation outcomes in analyses of other interventions [48]; the need to improve such reporting is well known in implementation science. When HIT adoption is not reported, it is difficult to assess an intervention’s population-level impact. In particular, if a limited number of potential users adopt an intervention, even when there is good reach and it is highly effective, population-level impacts may be low. Where adoption was reported, its rates were generally low (≤50%), underscoring the need for further research on improving the uptake of effective HIT [49]. When implementation barriers and facilitators to HIT adoption were reported, most related to inner context, outer context, and the nature of the innovation (including a given HIT tool’s function). Future research should assess which combination of these contextual factors is associated with the adoption of HIT with varied functions when used in different workflow steps (ie, panel management, point of care, and follow-up care). To further understand how contextual factors impact care teams’ adoption of HIT for cancer screening, there is also a need for more widespread reporting on practice type, which was rarely noted in the studies included here. Similarly, few studies reported on the sustainment of tool adoption. This evidence gap is seen throughout the implementation science literature [50]; improved knowledge of how to sustain the use of effective interventions is critical to maximizing their impact. Knowledge gap 3 describes the need for evidence on *how to improve* the adoption and maintenance of HIT-based interventions targeting cancer screening in primary care. We also posit that the lack of evidence on such interventions’ *reach* is relevant to how such interventions support *equity* in cancer screening, as discussed in knowledge gap 4.

### Knowledge Gap 3: Limited Evidence on Implementation Strategies That Support the Adoption of HIT Targeting Cancer Screening in Primary Care

A total of 24 studies (<25% of the included studies) reported on strategies used to support the adoption of HIT-based interventions, and few of these assessed the effectiveness of the strategies. This is complicated by the fact that in some cases a given HIT tool was considered the intervention or an intervention component, and in others, it was considered an implementation strategy for supporting the adoption of a clinical intervention. In the implementation science literature, the boundaries between clinical intervention and implementation intervention and between implementation intervention and implementation strategies are not always clear, adding complexity to this reporting.

Research is needed on how to support the adoption of HIT-based interventions targeting cancer screening using implementation strategies, how to use HIT as an implementation strategy, and what types of support strategies are used even in reports on HIT-based interventions’ impact. Reporting must strive to clearly differentiate between these approaches; the need for better reporting on implementation strategies is well known [51-53]. Although such reporting can be resource intensive, methods are emerging to facilitate it [40,54].

Research is also needed to specify how effectively different implementation strategies support the adoption of different HIT-based interventions in different care settings. Known barriers and facilitators to HIT adoption, in general, may also be impactful for HIT targeting cancer screening. For example, evidence indicates that barriers to HIT use include inadequate training for care teams on using EHR functions to their full potential [55-58]. Thus, effective implementation strategies for HIT targeting cancer prevention may involve training.

### Knowledge Gap 4: Limited Evidence on the Reach and Equitable Implementation of HIT for Cancer Screening in Primary Care

The equitable reach of HIT tools for cancer screening is poorly described. A few studies specifically focused on racial or ethnic minority groups; many were conducted in federally qualified health centers, which often serve racial or ethnic minority groups. Relevant data were reported in just 58.4% (59/101) of the studies included here. However, where such data were reported, eligible patients reached by the HIT interventions had a lower percentage of non-White patients than would be expected for the populations served, suggesting inequities in reach or underreporting. This is concerning, as racial disparities in cancer screening persist [59-61], and previous research found that interventions targeting breast or cervical cancer screening are less likely to target patients considered most at risk, for example, those in socioeconomically and racial or ethnic minoritized groups [5]. Findings from this scoping review underscore the need to understand potential drivers of these inequities (eg, design flaws in algorithms used to identify eligible patients and clinician bias in applying the HIT tool) and solutions to mitigate these inequities. One step toward addressing this inequity must involve improved reporting on how HIT is used in diverse patient populations. The well-documented need to improve reporting of race or ethnicity in health care [62] likely exacerbates the lack of reporting on the comparative reach of the tools included in this review among different groups. Another step toward equitable reach of HIT is understanding and addressing barriers to the inclusion of racial or ethnic minoritized patients in research on HIT adoption and impact. Future research on HIT adoption for cancer screening should explore strategies that support documentation, recruitment, and retention of racial or ethnic minoritized patients [63].

### Limitations

HIT-based interventions might be used to improve outcomes at each step of the cancer control continuum, such as risk assessment, prevention, detection, diagnosis, treatment, survivorship, and end-of-life care [15]. This review was limited to cancer screening. Furthermore, although breast, colorectal, and cervical cancer are highly prevalent cancers whose detection is in the purview of primary care, no other cancers recommended for screening in primary care (eg, lung cancer) were included; future research could assess whether the gaps identified in this study are seen for a broader set of cancers. This review was limited to US studies; therefore, the relevance of the findings is limited to the context of HIT policies and infrastructure as applicable to US primary care settings. Another potential limitation is that urban or rural status was defined based on what each study reported, and they may have used different methods for making this characterization.

In addition, the overlapping quality of some HIT characteristic categories (tool types and functions) made it difficult to execute related data charting. Similarly, content analysis of HIT functions was complicated when implementation strategies overlapped or when studies did not specify which cancers were targeted by the strategies. Our definition of effectiveness did not capture screening outcomes related to each clinical workflow (eg, an intervention using CDS for panel management showed improvements in colorectal cancer screening but did not clarify how improvements impacted screening initiation, completion, or follow-up care). Finally, we followed the PRISMA-ScR guidelines [34] to examine a broad array of literature to include studies that are heterogeneous in design and quality [64]. Although our search strategy followed an iterative process, it is possible that some relevant existing articles were not captured; we sought to mitigate this using a snowball search.

### Conclusions

In what is, to our knowledge, the first scoping review of the implementation of HIT-based interventions for cancer screening in primary care settings, we identified critical knowledge gaps. Little is known about the effectiveness of HIT-based interventions specifically targeting guideline-concordant breast and cervical cancer screening. Clarity is needed on the individual and combined effectiveness of HIT when integrated into a multicomponent intervention targeting cancer screening. Even less is known about how to enhance the adoption of cancer-targeted HIT in primary care. The potential for inequities in the reach of HIT for cancer screening remains underexplored. Research is necessary on implementation strategies to promote equitable access, ensuring that the potential benefits of HIT for population health are realized across diverse patient populations.

## Appendix

Multimedia Appendix 1 Database search strategy.

Multimedia Appendix 2 Gray literature search strategy.

Multimedia Appendix 3 Inclusion and exclusion criteria checklist.

Multimedia Appendix 4 Glossary of key terms.

Multimedia Appendix 5 Stage 5 procedures for qualitative content analysis.

Multimedia Appendix 6 References to the included studies (N=101).

Multimedia Appendix 7 Reporting status of health IT (HIT) effectiveness by cancer screening activities and HIT functions as represented in the included studies.

Multimedia Appendix 8 Reporting of health IT adoption as represented in the included studies.

Multimedia Appendix 9 Barriers and facilitators (with examples) of health IT adoption aligned with the Integrated Technology Implementation Model.

Multimedia Appendix 10 Expert Recommendations for Implementing Change implementation strategies used to support health IT adoption (n=24 studies).

Multimedia Appendix 11 PRISMA-ScR checklist.

## Abbreviations

CCIS: Consortium for Cancer Implementation Science
CDS: clinical decision support
EHR: electronic health record
ERIC: Expert Recommendations for Implementing Change
HIT: health IT
ITIM: Integrated Technology Implementation Model
PRISMA-ScR: Preferred Reporting Items for Systematic Reviews and Meta-Analyses extension for Scoping Reviews
RE-AIM: Reach, Effectiveness, Adoption, Implementation, and Maintenance
