# Supplementary material for: Implementation of Health IT for Cancer Screening in US Primary Care: Scoping Review
Source: JMIR Cancer. 2024 Apr 30;10:e49002. doi: 10.2196/49002 (PMC11094604; doi:10.2196/49002)
Supplement: Multimedia Appendix 1 [file cancer_v10i1e49002_app1.docx]

**Appendix 1. Database Search Strategy**

| **Database(s)** | **Search Strategy** | **Limits** |
| --- | --- | --- |
| Medline (MESH terms only) | MH "Neoplasms+/DI" OR MH "Early Detection of Cancer/IS/MT"  AND  ( MH "Primary Health Care+" OR MH "Physicians, Primary Care" OR MH "Primary Care Nursing" )  AND  ( MH "Implementation Science" OR MH "Technology+" OR MH "Point-of-Care Systems*" OR MH "Electronic Health Records+" OR MH "Patient Portals" OR MH "Decision Support Systems, Clinical" OR MH "Health Information Systems+" OR MH "Health Information Networks" OR MH "Telemedicine+" OR MH "Smartphone" ) | LIMITS: (YEAR) 2015-2021; (Lang) English |
|  |  |  |
| MEDLINE, CINAHL Plus & Web of Science  (Keywords only) | ( ( ("cancer*" OR "oncology" OR "neoplasm*" OR "carcinoma" OR "adenocarcinoma" OR "malignan*" OR "tumor" )) )  AND  ( ( ("breast" OR "colon" OR "rectal" OR "colorectal" OR "cervical" OR "cervix") ) )  AND  ( ( ("screen*" OR "early detection*" OR "secondary prevention" OR "cancer detection*" OR "cancer diagnos*" OR "colonoscopy" OR "sigmoidoscopy" OR "fecal occult blood test" OR "FOBT" OR "mammography" OR "pap test*" OR "pap smear*" OR "cervical smear" OR "DNA test*" OR "digital rectal exam*" OR "DRE" )) )  AND  ( ( ( "primary care" OR "primary health care" OR "primary healthcare" OR "primary community-based care" OR "primary clinical care" OR "general practice" OR "general practitioners") ) ) AND  ( ( ("implementation" OR "implement" OR "adopt" OR "sustain*" OR "quality improvement*" OR "disseminat*" OR "knowledge translation") ) )  AND  ( ( ("health information technolog*" OR "health IT" OR "electronic" OR "laptop*" OR "tablet*" OR "apps*" OR "software" OR "platform*" OR "digital health" OR "digital" OR "online" OR "internet" OR "web" OR "electronic medical record*" OR "electronic health record*" OR "personal medical record*" OR "personal health record*" OR "patient accessible record*" OR "patient portal*" OR "portal use" OR "use of portal" OR "web portal*" OR "website" OR "patient internet portal*" OR "Clinical decision support*" OR "clinical decision aid*" OR "decision support*" OR "clinical reminder*" OR "reminder system*" OR "computer assisted decision making" OR "computer assisted detection" OR "computer assisted diagnosis" OR "Electronic health information exchange*" OR "Health information exchange*" OR "health information interchange" OR "electronic document exchange*" OR "Information exchange*" OR "electronic data exchange*" OR "Telehealth" OR "telemedicine" OR "mhealth" OR "m-health" OR "information system*" OR "remote consult*" OR "remote patient monitoring" OR "RPM" OR "e-consult" OR "virtual" OR "ehealth" OR "e-health") ) ) | LIMITS: (YEAR) 2015-2021; (Lang) English |
|  |  |  |
| IEEE Xplore | ("Full Text & Metadata":"cancer*" OR "Full Text & Metadata":"oncology" OR "Full Text & Metadata":"neoplasm*" OR "Full Text & Metadata":"carcinoma" OR "Full Text & Metadata":"adenocarcinoma" OR "Full Text & Metadata":"malignan*" OR "Full Text & Metadata":"tumor") AND ("Full Text & Metadata":"breast" OR "Full Text & Metadata":"colon" OR "Full Text & Metadata":"rectal" OR "Full Text & Metadata":"colorectal" OR "Full Text & Metadata":"cervical" OR "Full Text & Metadata":"cervix") AND ("Full Text & Metadata":"screen*" OR "Full Text & Metadata":"early detection*" OR "Full Text & Metadata":"secondary prevention" OR "Full Text & Metadata":"cancer detection*" OR "Full Text & Metadata":"cancer diagnos*" OR "Full Text & Metadata":"DNA test*" OR "Full Text & Metadata":"digital rectal exam*") AND ("Full Text & Metadata":"primary care" OR "Full Text & Metadata":"primary health care" OR "Full Text & Metadata":"primary healthcare" OR "Full Text & Metadata":"primary community-based care" OR "Full Text & Metadata":"primary clinical care" OR "Full Text & Metadata":"general practice" OR "Full Text & Metadata":"general practitioners") AND ("Full Text & Metadata":"implementation" OR "Full Text & Metadata":"implement" OR "Full Text & Metadata":"adopt" OR "Full Text & Metadata":"sustain*" OR "Full Text & Metadata":"quality improvement*" OR "Full Text & Metadata":"disseminat*" OR "Full Text & Metadata":"knowledge translation") AND ("Full Text & Metadata":"health information technolog*" OR "Full Text & Metadata":"health IT" OR "Full Text & Metadata":"electronic" OR "Full Text & Metadata":"apps*" OR "Full Text & Metadata":"software" OR "Full Text & Metadata":"ehealth" OR "Full Text & Metadata":"mhealth") | LIMITS: (YEAR) 2015-2021; (can't limit by lang) |
