# Supplementary material for: Implementation of Health IT for Cancer Screening in US Primary Care: Scoping Review
Source: JMIR Cancer. 2024 Apr 30;10:e49002. doi: 10.2196/49002 (PMC11094604; doi:10.2196/49002)
Supplement: Multimedia Appendix 2 [file cancer_v10i1e49002_app2.docx]

Appendix 2. Gray Literature Search Strategy

The following search terms were used for each source provided below: cancer screening, cancer, colorectal cancer, breast cancer, cervical cancer, health information technology. Additionally, when search terms were not used, all webpages via the websites provided in this table were reviewed for relevant references.

| **Source** | **Website Link** |
| --- | --- |
| National Cancer Institute | <https://www.cancer.gov/publications> |
| Agency for Healthcare Research and Quality (AHRQ) | <https://digital.ahrq.gov/ahrq-funded-projects/search> |
| Centers for Disease Control and Prevention (CDC) | 1. <https://www.cdc.gov/cancer/dcpc/resources/> 2. <https://www.cdc.gov/cancer/dcpc/about/programs.htm> 3. <https://www.cdc.gov/cancer/dcpc/about/campaigns.htm> 4. <https://www.cdc.gov/cancer/dcpc/about/initiatives.htm> |
| Centers for Medicare & Medicaid Services (CMS) | <https://www.cms.gov/> |
| Office of the National Coordinator for Health Information Technology (ONC) | <https://www.healthit.gov/topic/health-it-resources> |
| Health Resources and Services Administration | <https://www.hrsa.gov/> |
| U.S. Preventive Services Task Force (USPSTF) | <https://www.uspreventiveservicestaskforce.org/uspstf/> |
| Clinicaltrials.gov | <https://clinicaltrials.gov/> |
| American Cancer Society | <https://www.cancer.org/> |
| RAND Corporation | <https://www.rand.org/> |
| ProQuest Dissertation | <https://about.proquest.com/en/dissertations/> |
| George Washington University Cancer Center | <https://smhs.gwu.edu/cancercontroltap/> |
| American Society of Clinical Oncology | 1. <https://www.asco.org/> 2. <https://conferences.asco.org/am/abstracts> |
| American Medical Informatics Association | <https://www.amia.org/cic2021> |
