# Supplementary material for: Implementation of Health IT for Cancer Screening in US Primary Care: Scoping Review
Source: JMIR Cancer. 2024 Apr 30;10:e49002. doi: 10.2196/49002 (PMC11094604; doi:10.2196/49002)
Supplement: Multimedia Appendix 3 [file cancer_v10i1e49002_app3.docx]

Appendix 3. Inclusion and Exclusion Criteria Checklist

1. **Is this a U.S-based reference in English language, published in January 2015-June 2021?**
   - Yes/Don’t Know: move on to question #2
   - No: Stop screening and move the reference to the appropriate “Exclude” folder
2. **Is the setting primary care?**
   - Yes/Don’t Know: move on to question #3
   - No: Stop screening and move the reference to the appropriate “Exclude” folder
3. **Is the topic focus secondary prevention of cancer (cancer screening), rather than survivorship care?**
   - Yes/Don’t Know: move on to question #4
   - No: Stop screening and move the reference to the appropriate “Exclude” folder
4. **Is HIT used to support the secondary prevention (cancer screening) activities described in this reference?** Refer to *Table 1 in Key Terms* section for examples of how HIT is used to support secondary prevention of cancer (cancer screening). Please note, the reference can either discuss the HIT tool(s) or implementation strategies for its adoption.
   - Yes/Don’t Know: move on to question #5
   - No: Stop screening and move the reference to the appropriate “Exclude” folder
5. **Do the secondary prevention (cancer screening) activities involve: 1) identification of patients who are due for cancer screening in 1a) panel management, or 1b) at the point of care, 2) electronic acquisition of prior cancer screening results, 3) follow-up on cancer screening referral status or 4) follow-up care (including scheduling next test and/or oncology referral) for positive cancer screening results?**
   - Yes/Don’t Know: move on to question #6
   - No: Stop screening and move the reference to the appropriate “Exclude” folder
6. **Do the secondary prevention (cancer screening) activities from Question #5 target guideline-concordant screening for breast, colorectal, and/or cervical cancer?** Refer to *Guideline-Concordant Cancer Screening Procedures,* for a complete list of screening procedures that meet national guidelines for secondary prevention (screening) of breast, colorectal, and cervical cancer.
   - Yes/Don’t Know: move the reference to the appropriate “Include” folder
   - No: Stop screening and move the reference to the appropriate “Exclude” folder

**TITLE/ABSTRACT REVIEW**

- **INCLUDE FOR FULL-TEXT REVIEW (FTR):** If you answered ‘Yes’ to all questions or ‘Don’t know’ to at least one of these questions, move reference to **“Include (FTR)” folder.**
- **EXCLUDE FOR FTR:** If you answered ‘No’ to any question, move reference to **“Exclude (FTR)” folder.**
- **UNSURE FOR FTR:** If you answered ‘Yes’ to all questions or ‘Don’t know’ to at least one of these questions, but still remain unsure if this reference should be included for full-text review, move reference to **“Unsure (FTR)” folder.**

**FULL-TEXT REVIEW (FTR)**

- **INCLUDE FOR DATA CHARTING (DC):** If you answered ‘Yes’ to all questions, move reference to “**Include (DC)” folder.**
- **EXCLUDE FOR DC:** If you answered ‘No’ to any of the questions stop the review and move reference to **“Exclude (DC)” folder.**
- **UNSURE FOR DC:** If you answered ‘Yes’ to all questions or ‘Don’t know’ to at least one of these questions, but still remain unsure if this reference should be included for data charting, move reference to **“Unsure (DC)” folder.**
