# Supplementary material for: Implementation of Health IT for Cancer Screening in US Primary Care: Scoping Review
Source: JMIR Cancer. 2024 Apr 30;10:e49002. doi: 10.2196/49002 (PMC11094604; doi:10.2196/49002)
Supplement: Multimedia Appendix 4 [file cancer_v10i1e49002_app4.docx]

**Appendix 4. Glossary of Key Terms**

| **Term** | **Definition** |
| --- | --- |
| **Health Information Technology (HIT)** | “Health IT, shorthand for ‘health information technology,’ is a broad concept that encompasses an array of technologies. Health IT is the use of computer hardware, software, or infrastructure to record, store, protect, and retrieve clinical, administrative, or financial information. Health IT can include: electronic health records, personal health records, electronic medical records, electronic prescribing (e-Prescribing). The overarching purpose of Health IT is to use health care information, data, and knowledge for communication and decision making.” ^1^ |
| **Primary Care** | Primary care is the provision of integrated, accessible health care services by clinicians who are accountable for addressing a large majority of personal health care needs, developing a sustained partnership with patients, and practicing in the context of family and community.^2^ The care provided by certain clinicians—Some proposed legislation, for example, lists the medical specialties of primary care as family medicine, general internal medicine, general pediatrics, and obstetrics and gynecology. Some experts and groups have included nurse practitioners and physician assistants.^3^ |
| **Secondary Prevention of Cancer** | “Secondary prevention is that set of interventions leading to the discovery and control of cancerous or precancerous processes while localized, i.e., screening, early detection, and effective treatment.” ^4^ |
| **Cancer Screening** | “Checking for disease when there are no symptoms. Since screening may find diseases at an early stage, there may be a better chance of curing the disease.” Examples of cancer screening tests are the mammogram (for breast cancer), colonoscopy (for colon cancer), and the Pap test and HPV tests (for cervical cancer). Screening can also include doing a genetic test to check for a person’s risk of developing an inherited disease.” ^5^ |
| **Survivorship Care** | “In cancer, survivorship focuses on the health and well-being of a person with cancer from the time of diagnosis until the end of life. This includes the physical, mental, emotional, social, and financial effects of cancer that begin at diagnosis and continue through treatment and beyond. The survivorship experience also includes issues related to follow-up care (including regular health and wellness checkups), late effects of treatment, cancer recurrence, second cancers, and quality of life.” ^6^ |
| **Implementation Strategies** | “Methods or techniques used to enhance the adoption, implementation and sustainability of a clinical program or practice.” ^7^ In the context of this scoping review, implementation strategies can be the HIT tools used to enhance guideline-concordant cancer screening for breast, colorectal, and/or cervical cancer, or implementation strategies can be the approaches used to improve adoption of HIT tools to support guideline-concordant cancer screening procedures. |
| ***Cancer Screening Activities Supported by HIT*** |  |
| **Electronic acquisition of prior cancer screening results** | Activities involved with electronically attaining prior cancer screening results or sharing patient data. |
| **Panel management** | “A panel is a list of patients assigned to each care team in the practice. The care team (e.g., a physician, a medical assistant, and a health educator) is responsible for preventive care, disease management, and acute care for all the patients on its panel. This means that a patient will have the opportunity to receive care from the same clinician and his or her care team. Panel management, also known as population management, is a proactive approach to health care. ‘Population’ means the panel of patients associated with a provider or care team. Population-based care means that the care team is concerned with the health of the entire population of its patients, not just those who come in for visits. For example, a care team with a panel of 1,500 patients would be concerned about the health care needs of the entire 1,500. The team would work on anticipating and planning for this care proactively (in advance) rather than reactively (when the patient shows up for a visit and requests care).” ^8^ |
| **Point of care** | Patient services delivered in-person and/or by telehealth/telemedicine. |
| **Follow-up care for positive cancer screening results** | Immediate follow-up care activities (including scheduling next test and/or oncology referral) for positive cancer screening results, to help patient begin appropriate care plan per current guidelines. |
| **Follow-up on cancer screening referral status** | Activities involved with identifying if the patient completed/failed to complete referral for cancer screening. |
| **Other/Unclear** | Any cancer screening activities that are not captured in the aforementioned five groups. |
| ***HIT Sources*** |  |
| **EHR-based** | Electronic health record (EHR) systems, or tools integrated in the EHR system, to communicate health information to providers or patients. |
| **Web-based** | Online applications designed to communicate health information to providers or patients through the internet. |
| **Other/Unclear** | Tools that did not fall into either category or were not clearly EHR-based or Web-based (according to the descriptions above). |
| ***HIT Functions*** |  |
| **Clinical Decision Support (CDS) - Point of Care (POC)** | To support the care team (clinicians and non-clinical staff) with the identification of patients in need of cancer screening at the point of care (e.g., during the patient encounter).* |
| **Clinical Decision Support (CDS) - panel management/outreach** | During panel management / outreach 1) support the care team with the identification patients in need of cancer screening; and 2) provide patient reminders and support patient navigation.* |
| **Risk Identification** | Use EHR data to identify high-risk patients due / past due for screening and / or in need of follow-up care, for outreach purposes.* |
| **Patient Decision Aid** | Electronic tools to improve patient awareness of importance of getting screened / support shared decision-making.* |
| **Provider Assessment and Feedback** | “Evaluate provider performance in offering and/or delivering screening to patients (assessment) and present providers with information about their performance in providing screening services (feedback). Feedback may describe the performance of a group of providers or an individual provider and may be compared with a goal or standard.”^9^ |
| **Tracking Patient Adherence** | Use EHR data to monitor patient adherence to recommended care plan / follow-up care.* |

**Footnotes:**

**1 Sources: Office of the National Coordinator for Health Information Technology (ONC). Available here:** [**https://www.healthit.gov/faq/what-health-it**](https://www.healthit.gov/faq/what-health-it)**; Thompson T, Brailer D. Health IT Strategic Framework. Washington: US Department of Health and Human Services; 2004. Available here:** [**http://www.providersedge.com/ehdocs/ehr_articles/the_decade_of_hit-delivering_customer-centric_and_info-rich_hc.pdf**](http://www.providersedge.com/ehdocs/ehr_articles/the_decade_of_hit-delivering_customer-centric_and_info-rich_hc.pdf)

**2 Source:** [**https://www.nap.edu/read/5152/chapter/4#31**](https://www.nap.edu/read/5152/chapter/4#31)

**3 Source: OTA, 1986; Pew Health Professions Commission, 1994**

**4 Source: Spratt JS. The primary and secondary prevention of cancer. J Surg Oncol. 1981;18(3):219-230. doi:10.1002/jso.2930180302**

**5 Source: Centers for Disease Control and Prevention (CDC). Cancer. Available here:** [**https://www.cdc.gov/cancer/dcpc/prevention/screening.htm**](https://www.cdc.gov/cancer/dcpc/prevention/screening.htm)

**6 Source: NCI. NCI Dictionary of Cancer Terms. Available here:** [**https://www.cancer.gov/publications/dictionaries/cancer-terms/def/survivorship**](https://www.cancer.gov/publications/dictionaries/cancer-terms/def/survivorship)

**7Sources: Powell, B.J., Waltz, T.J., Chinman, M.J. et al. A refined compilation of implementation strategies: results from the Expert Recommendations for Implementing Change (ERIC) project. Implementation Sci 10, 21 (2015). https://doi.org/10.1186/s13012-015-0209-1; Proctor EK, Powell BJ, McMillen JC. Implementation strategies: recommendations for specifying and reporting. Implement Sci. 2013;8:139. Published 2013 Dec 1. doi:10.1186/1748-5908-8-139**

**8 Source: Agency for Healthcare Research and Quality (AHRQ). Practice Facilitation Handbook. Available here:** [**https://www.ahrq.gov/ncepcr/tools/pf-handbook/mod20.html**](https://www.ahrq.gov/ncepcr/tools/pf-handbook/mod20.html)

**9 Source: CDC’s The Guide to Community Preventive Services:** [**https://www.thecommunityguide.org/findings/cancer-screening-provider-assessment-and-feedback-cervical-cancer.html**](https://www.thecommunityguide.org/findings/cancer-screening-provider-assessment-and-feedback-cervical-cancer.html)

***: Sourced from content analysis of HIT functions conducted as part of this study**
