# Supplementary material for: Implementation of Health IT for Cancer Screening in US Primary Care: Scoping Review
Source: JMIR Cancer. 2024 Apr 30;10:e49002. doi: 10.2196/49002 (PMC11094604; doi:10.2196/49002)
Supplement: Multimedia Appendix 5 [file cancer_v10i1e49002_app5.docx]

Appendix 5. Stage 5 Procedures for Qualitative Content Analysis

First, the first and senior authors independently analyzed charted text data for HIT tools, functions, and cancer screening activities supported by HIT based on an initial list of concepts developed by the first and senior authors. Through repeated examination of the charted free text data for these select HIT characteristics, an inductive approach was used to modify the initial concepts based on emergent patterns and relationships between HIT functions and cancer activities supported by HIT.

A similar hybrid deductive-inductive approach was used to analyze free text for implementation strategies, barriers, and facilitators. Since implementation strategies were charted deductively based on the ERIC compilation, two authors used an inductive approach to identify emergent themes in free text data for each implementation strategy reporting domain (e.g., actor, target of action). Similarly, since barriers and facilitators were charted deductively using ITIM framework, free text data was independently analyzed by the first author using an inductive approach to identify emergent themes for each ITIM construct.

Second, charted data that did not clearly fit in predefined categories for reference, practice, and HIT characteristics were charted as ‘other.’ Two authors used an inductive approach to identify thematic concepts for this free text data. Throughout these analyses, four authors met to cross check coding, resolve discrepancies, and to reach consensus on concept names and meanings to summarize the data.
