# Supplementary material for: Implementation of Health IT for Cancer Screening in US Primary Care: Scoping Review
Source: JMIR Cancer. 2024 Apr 30;10:e49002. doi: 10.2196/49002 (PMC11094604; doi:10.2196/49002)
Supplement: Multimedia Appendix 6 [file cancer_v10i1e49002_app6.docx]

Appendix 6. References to Included Studies (N =101)

| **Citations** | **Cancer Screening Type** | **Publication Year** |
| --- | --- | --- |
| 1. Coronado GD, Petrik AF, Vollmer WM, et al. Effectiveness of a Mailed Colorectal Cancer Screening Outreach Program in Community Health Clinics: The STOP CRC Cluster Randomized Clinical Trial. *JAMA Intern Med*. 2018;178(9):1174-1181. doi:[10.1001/jamainternmed.2018.3629](https://doi.org/10.1001/jamainternmed.2018.3629) | colorectal | 2018 |
| 1. Kelsey EA, Njeru JW, Chaudhry R, Fischer KM, Schroeder DR, Croghan IT. Understanding User Acceptance of Clinical Decision Support Systems to Promote Increased Cancer Screening Rates in a Primary Care Practice. *Journal of primary care & community health*. 2020;11:2150132720958832. doi:[10.1177/2150132720958832](https://doi.org/10.1177/2150132720958832) | breast, colorectal, cervical | 2020 |
| 1. Kemper KE, Glaze BL, Eastman CL, et al. Effectiveness and cost of multilayered colorectal cancer screening promotion interventions at federally qualified health centers in Washington State. *Cancer*. 2018;124(21):4121-4129. doi:[10.1002/cncr.31693](https://doi.org/10.1002/cncr.31693) | colorectal | 2018 |
| 1. Kim KE, Tangka FKL, Jayaprakash M, et al. Effectiveness and Cost of Implementing Evidence-Based Interventions to Increase Colorectal Cancer Screening Among an Underserved Population in Chicago. *Health Promot Pract*. 2020;21(6):884-890. doi:[10.1177/1524839920954162](https://doi.org/10.1177/1524839920954162) | colorectal | 2020 |
| 1. J. Wood, K. Crew, R. Kukafka, J. Finkelstein. A Comprehensive Informatics Framework to Increase Breast Cancer Risk Assessment and Chemoprevention in the Primary Care Setting. In: *2016 IEEE International Conference on Healthcare Informatics (ICHI)*. ; 2016:293-296. doi:[10.1109/ICHI.2016.41](https://doi.org/10.1109/ICHI.2016.41) | breast | 2016 |
| 1. Kim J, Young L, Bekmuratova S, et al. Promoting colorectal cancer screening through a new model of delivering rural primary care in the USA: a qualitative study. *RURAL AND REMOTE HEALTH*. 2017;17(1). <https://pubmed.ncbi.nlm.nih.gov/28355878/> | colorectal | 2017 |
| 1. Kim J, Wang H, Young L, et al. An Examination of Multilevel Factors Influencing Colorectal Cancer Screening in Primary Care Accountable Care Organization Settings: A Mixed-Methods Study. *JOURNAL OF PUBLIC HEALTH MANAGEMENT AND PRACTICE*. 2019;25(6, SI):562-570. doi:[10.1097/PHH.0000000000000837](https://doi.org/10.1097/PHH.0000000000000837) | colorectal | 2019 |
| 1. Schlauderaff P, Baldino T, Graham KC, et al. Colorectal cancer screening in a rural US population. *INTERNATIONAL JOURNAL OF HEALTH GOVERNANCE*. 2017;22(4):283-291. doi:[10.1108/IJHG-05-2017-0021](https://doi.org/10.1108/IJHG-05-2017-0021) | colorectal | 2017 |
| 1. Lara CL, Means KL, Morwood KD, et al. Colorectal cancer screening interventions in 2 health care systems serving disadvantaged populations: Screening uptake and cost-effectiveness. *Cancer*. 2018;124(21):4130-4136. doi:[10.1002/cncr.31691](https://doi.org/10.1002/cncr.31691) | colorectal | 2018 |
| 1. Kruse-Diehr AJ, Dignan M, Cromo M, et al. Building Cancer Prevention and Control Research Capacity in Rural Appalachian Kentucky Primary Care Clinics During COVID-19: Development and Adaptation of a Multilevel Colorectal Cancer Screening Project. *JOURNAL OF CANCER EDUCATION*. 2021; doi:[10.1007/s13187-021-01972-w](https://doi.org/10.1007/s13187-021-01972-w) | colorectal | 2021 |
| 1. Levin TR, Corley DA, Jensen CD, et al. Effects of Organized Colorectal Cancer Screening on Cancer Incidence and Mortality in a Large Community-Based Population. *Gastroenterology*. 2018;155(5):1383-1391.e5. doi:[10.1053/j.gastro.2018.07.017](https://doi.org/10.1053/j.gastro.2018.07.017) | colorectal | 2018 |
| 1. Lamanna A, Sheaffer H, Guerra C, Kochman M. Colorectal Cancer Screening Navigation for the Underserved: Experience of an Urban Program. *Gastroenterol Hepatol (N Y)*. 2016;12(9):547-551. | colorectal | 2016 |
| 1. Singal AG, Gupta S, Skinner CS, et al. Effect of Colonoscopy Outreach vs Fecal Immunochemical Test Outreach on Colorectal Cancer Screening Completion: A Randomized Clinical Trial. *JAMA*. 2017;318(9):806-815. doi:[10.1001/jama.2017.11389](https://doi.org/10.1001/jama.2017.11389) | colorectal | 2017 |
| 1. Kukafka R, Yi H, Xiao T, et al. Why Breast Cancer Risk by the Numbers Is Not Enough: Evaluation of a Decision Aid in Multi-Ethnic, Low-Numerate Women. *Journal of medical Internet research*. 2015;17(7):e165. doi:[10.2196/jmir.4028](https://doi.org/10.2196/jmir.4028) | breast | 2015 |
| 1. Kim K, Polite B, Hedeker D, et al. Implementing a multilevel intervention to accelerate colorectal cancer screening and follow-up in federally qualified health centers using a stepped wedge design: a study protocol. *Implement Sci*. 2020;15(1):96. doi:[10.1186/s13012-020-01045-4](https://doi.org/10.1186/s13012-020-01045-4) | colorectal | 2020 |
| 1. Krist AH, Woolf SH, Hochheimer C, et al. Harnessing Information Technology to Inform Patients Facing Routine Decisions: Cancer Screening as a Test Case. *ANNALS OF FAMILY MEDICINE*. 2017;15(3):217-224. doi:[10.1370/afm.2063](https://doi.org/10.1370/afm.2063) | breast, colorectal | 2017 |
| 1. Klein KA, Watson L, Ash JS, Eden KB. Evaluation of risk communication in a mammography patient decision aid. *Patient Educ Couns*. 2016;99(7):1240-1248. doi:[10.1016/j.pec.2016.02.013](https://doi.org/10.1016/j.pec.2016.02.013) | breast | 2016 |
| 1. Levy DE, Munshi VN, Ashburner JM, Zai AH, Grant RW, Atlas SJ. Health IT-assisted population-based preventive cancer screening: a cost analysis. *Am J Manag Care*. 2015;21(12):885-891. | breast, colorectal, cervical | 2015 |
| 1. Liles EG, Schneider JL, Feldstein AC, et al. Implementation challenges and successes of a population-based colorectal cancer screening program: a qualitative study of stakeholder perspectives. *IMPLEMENTATION SCIENCE*. 2015;10. doi:[10.1186/s13012-015-0227-z](https://doi.org/10.1186/s13012-015-0227-z) | colorectal | 2015 |
| 1. Kruse-Diehr AJ, Oliveri JM, Vanderpool RC, et al. Development of a multilevel intervention to increase colorectal cancer screening in Appalachia. *Implementation science communications*. 2021;2(1):51. doi:[10.1186/s43058-021-00151-8](https://doi.org/10.1186/s43058-021-00151-8) | colorectal | 2021 |
| 1. Luckmann R, White MJ, Costanza ME, et al. Implementation and process evaluation of three interventions to promote screening mammograms delivered for 4 years in a large primary care population. *TRANSLATIONAL BEHAVIORAL MEDICINE*. 2017;7(3):547-556. doi:[10.1007/s13142-017-0497-x](https://doi.org/10.1007/s13142-017-0497-x) | breast | 2017 |
| 1. Fedewa SA, Corley DA, Jensen CD, et al. Colorectal Cancer Screening Initiation After Age 50 Years in an Organized Program. *Am J Prev Med*. 2017;53(3):335-344. doi:[10.1016/j.amepre.2017.02.018](https://doi.org/10.1016/j.amepre.2017.02.018) | colorectal | 2017 |
| 1. MacLaughlin KL, Kessler ME, Elayavilli RK, et al. Impact of Patient Reminders on Papanicolaou Test Completion for High-Risk Patients Identified by a Clinical Decision Support System. *JOURNAL OF WOMENS HEALTH*. 2018;27(5):569-574. doi:[10.1089/jwh.2017.6667](https://doi.org/10.1089/jwh.2017.6667) | cervical | 2018 |
| 1. Phillips L, Hendren S, Humiston S, Winters P, Fiscella K. Improving breast and colon cancer screening rates: a comparison of letters, automated phone calls, or both. *J Am Board Fam Med*. 2015;28(1):46-54. doi:[10.3122/jabfm.2015.01.140174](https://doi.org/10.3122/jabfm.2015.01.140174) | breast, colorectal | 2015 |
| 1. Modica C, Lewis JH, Bay C. Colorectal Cancer: Applying the Value Transformation Framework to increase the percent of patients receiving screening in Federally Qualified Health Centers. *PREVENTIVE MEDICINE REPORTS*. 2019;15. doi:[10.1016/j.pmedr.2019.100894](https://doi.org/10.1016/j.pmedr.2019.100894) | colorectal | 2019 |
| 1. Pierre-Joseph N. A Single-Arm Proof of Concept, Open Trial Clinical Study Investigating the Feasibility and Efficacy of Integrating Behavioral and Mobile Health Educational Interventions for Primary and Secondary Prevention in the Primary Care Setting. clinicaltrials.gov; 2017. <https://clinicaltrials.gov/ct2/show/NCT03033550> | cervical | 2017 |
| 1. Ravikumar KE, MacLaughlin KL, Scheitel MR, et al. Improving the Accuracy of a Clinical Decision Support System for Cervical Cancer Screening and Surveillance. *Appl Clin Inform*. 2018;9(1):62-71. doi:[10.1055/s-0037-1617451](https://doi.org/10.1055/s-0037-1617451) | cervical | 2018 |
| 1. Rawl SM, Christy SM, Perkins SM, et al. Computer-tailored intervention increases colorectal cancer screening among low-income African Americans in primary care: Results of a randomized trial. *Preventive medicine*. 2021;145:106449. doi:[10.1016/j.ypmed.2021.106449](https://doi.org/10.1016/j.ypmed.2021.106449) | colorectal | 2021 |
| 1. Saman DM, Walton KM, Harry ML, et al. Understanding primary care providers’ perceptions of cancer prevention and screening in a predominantly rural healthcare system in the upper Midwest. *BMC health services research*. 2019;19(1):1019. doi:[10.1186/s12913-019-4872-9](https://doi.org/10.1186/s12913-019-4872-9) | breast, colorectal, cervical | 2019 |
| 1. Schapira MM, Sprague BL, Klabunde CN, et al. Inadequate Systems to Support Breast and Cervical Cancer Screening in Primary Care Practice. *Journal of general internal medicine*. 2016;31(10):1148-1155. doi:[10.1007/s11606-016-3726-y](https://doi.org/10.1007/s11606-016-3726-y) | breast, cervical | 2016 |
| 1. Myers RE, Stello B, Daskalakis C, et al. Decision Support and Navigation to Increase Colorectal Cancer Screening Among Hispanic Patients. *CANCER EPIDEMIOLOGY BIOMARKERS & PREVENTION*. 2019;28(2):384-391. doi:[10.1158/1055-9965.EPI-18-0260](https://doi.org/10.1158/1055-9965.EPI-18-0260) | colorectal | 2019 |
| 1. Militello LG, Saleem JJ, Borders MR, et al. Designing Colorectal Cancer Screening Decision Support: A Cognitive Engineering Enterprise. *J Cogn Eng Decis Mak*. 2016;10(1):74-90. doi:[10.1177/1555343416630875](https://doi.org/10.1177/1555343416630875) | colorectal | 2016 |
| 1. Navarrete-Pak J. Improving colorectal cancer screening in primary care. *Improving Colorectal Cancer Screening in Primary Care*. 2016;(Ph.D.):1-1. | colorectal | 2016 |
| 1. Percac-Lima S, Ashburner JM, Zai AH, et al. Patient Navigation for Comprehensive Cancer Screening in High-Risk Patients Using a Population-Based Health Information Technology System: A Randomized Clinical Trial. *JAMA Intern Med*. 2016;176(7):930-937. doi:[10.1001/jamainternmed.2016.0841](https://doi.org/10.1001/jamainternmed.2016.0841) | breast, colorectal, cervical | 2016 |
| 1. Muller CJ, Robinson RF, Smith JJ, et al. Text message reminders increased colorectal cancer screening in a randomized trial with Alaska Native and American Indian people. *Cancer*. 2017;123(8):1382-1389. doi:[10.1002/cncr.30499](https://doi.org/10.1002/cncr.30499) | colorectal | 2017 |
| 1. Patel MS, Volpp KG, Small DS, et al. Using active choice within the electronic health record to increase physician ordering and patient completion of high-value cancer screening tests. *Healthc (Amst)*. 2016;4(4):340-345. doi:[10.1016/j.hjdsi.2016.04.005](https://doi.org/10.1016/j.hjdsi.2016.04.005) | breast, colorectal | 2016 |
| 1. Joseph DA. Use of Evidence-Based Interventions to Address Disparities in Colorectal Cancer Screening. *MMWR Suppl*. 2016;65. doi:[10.15585/mmwr.su6501a5](https://doi.org/10.15585/mmwr.su6501a5) | colorectal | 2016 |
| 1. Elliott TE, O’Connor PJ, Asche SE, et al. Design and rationale of an intervention to improve cancer prevention using clinical decision support and shared decision making: A clinic-randomized trial. *CONTEMPORARY CLINICAL TRIALS*. 2021;102. doi:[10.1016/j.cct.2021.106271](https://doi.org/10.1016/j.cct.2021.106271) | breast, colorectal, cervical | 2021 |
| 1. Bowen ME, Bhat D, Fish J, et al. Improving Performance on Preventive Health Quality Measures Using Clinical Decision Support to Capture Care Done Elsewhere and Patient Exceptions. *American journal of medical quality : the official journal of the American College of Medical Quality*. 2018;33(3):237-245. doi:[10.1177/1062860617732830](https://doi.org/10.1177/1062860617732830) | breast, colorectal, cervical | 2018 |
| 1. Coronado GD, Schneider JL, Petrik A, Rivelli J, Taplin S, Green BB. Implementation successes and challenges in participating in a pragmatic study to improve colon cancer screening: perspectives of health center leaders. *TRANSLATIONAL BEHAVIORAL MEDICINE*. 2017;7(3):557-566. doi:[10.1007/s13142-016-0461-1](https://doi.org/10.1007/s13142-016-0461-1) | colorectal | 2017 |
| 1. DeGroff A. Increasing Colorectal Cancer Screening in Health Care Systems Using Evidence-Based Interventions. *Prev Chronic Dis*. 2018;15. doi:[10.5888/pcd15.180029](https://doi.org/10.5888/pcd15.180029) | colorectal | 2018 |
| 1. Haas JS, Baer HJ, Eibensteiner K, et al. A Cluster Randomized Trial of a Personalized Multi-Condition Risk Assessment in Primary Care. *Am J Prev Med*. 2017;52(1):100-105. doi:[10.1016/j.amepre.2016.07.013](https://doi.org/10.1016/j.amepre.2016.07.013) | breast, colorectal | 2017 |
| 1. Eden KB, Scariati P, Klein K, et al. Mammography Decision Aid Reduces Decisional Conflict for Women in Their Forties Considering Screening. *J Womens Health (Larchmt)*. 2015;24(12):1013-1020. doi:[10.1089/jwh.2015.5256](https://doi.org/10.1089/jwh.2015.5256) | breast | 2015 |
| 1. DeGroff A, Schroy PC, Morrissey KG, et al. Patient Navigation for Colonoscopy Completion: Results of an RCT. *Am J Prev Med*. 2017;53(3):363-372. doi:[10.1016/j.amepre.2017.05.010](https://doi.org/10.1016/j.amepre.2017.05.010) | colorectal | 2017 |
| 1. Dyer KE, Shires DA, Flocke SA, et al. Patient-Reported Needs Following a Referral for Colorectal Cancer Screening. *AMERICAN JOURNAL OF PREVENTIVE MEDICINE*. 2019;56(2):271-280. doi:[10.1016/j.amepre.2018.08.017](https://doi.org/10.1016/j.amepre.2018.08.017) | colorectal | 2019 |
| 1. Daly JM, Levy BT, Moss CA, Bay CP. System Strategies for Colorectal Cancer Screening at Federally Qualified Health Centers. *Am J Public Health*. 2015;105(1):212-219. doi:[10.2105/AJPH.2013.301790](https://doi.org/10.2105/AJPH.2013.301790) | colorectal | 2015 |
| 1. Eden KB, Ivlev I, Bensching KL, et al. Use of an Online Breast Cancer Risk Assessment and Patient Decision Aid in Primary Care Practices. *Journal of Women’s Health (15409996)*. 2020;29(6):763-769. doi:[10.1089/jwh.2019.8143](https://doi.org/10.1089/jwh.2019.8143) | breast | 2020 |
| 1. Hsiang EY, Mehta SJ, Small DS, et al. Association of an Active Choice Intervention in the Electronic Health Record Directed to Medical Assistants With Clinician Ordering and Patient Completion of Breast and Colorectal Cancer Screening Tests. *JAMA NETWORK OPEN*. 2019;2(11). doi:[10.1001/jamanetworkopen.2019.15619](https://doi.org/10.1001/jamanetworkopen.2019.15619) | breast, colorectal | 2019 |
| 1. Schwartz PH, Imperiale TF, Perkins SM, Schmidt KK, Althouse S, Rawl SM. Impact of including quantitative information in a decision aid for colorectal cancer screening: A randomized controlled trial. *Patient education and counseling*. 2019;102(4):726-734. doi:[10.1016/j.pec.2018.11.010](https://doi.org/10.1016/j.pec.2018.11.010) | colorectal | 2019 |
| 1. Harry ML, Truitt AR, Saman DM, et al. Barriers and facilitators to implementing cancer prevention clinical decision support in primary care: a qualitative study. *BMC Health Serv Res*. 2019;19(1):534. doi:[10.1186/s12913-019-4326-4](https://doi.org/10.1186/s12913-019-4326-4) | breast, colorectal, cervical | 2019 |
| 1. Issaka RB, Akinsoto NO, Strait E, Chaudhari V, Flum DR, Inadomi JM. Effectiveness of a mailed fecal immunochemical test outreach: a Medicare Advantage pilot study. *THERAPEUTIC ADVANCES IN GASTROENTEROLOGY*. 2020;13. doi:[10.1177/1756284820945388](https://doi.org/10.1177/1756284820945388) | colorectal | 2020 |
| 1. Harry ML, Saman DM, Truitt AR, et al. Pre-implementation adaptation of primary care cancer prevention clinical decision support in a predominantly rural healthcare system. *BMC MEDICAL INFORMATICS AND DECISION MAKING*. 2020;20(1). doi:[10.1186/s12911-020-01136-8](https://doi.org/10.1186/s12911-020-01136-8) | breast, colorectal, cervical | 2020 |
| 1. Hardin V, Tangka FKL, Wood T, et al. The Effectiveness and Cost to Improve Colorectal Cancer Screening in a Federally Qualified Homeless Clinic in Eastern Kentucky. *Health Promot Pract*. 2020;21(6):905-909. doi:[10.1177/1524839920954165](https://doi.org/10.1177/1524839920954165) | colorectal | 2020 |
| 1. Inadomi JM, Issaka RB, Green BB. What Multilevel Interventions Do We Need to Increase the Colorectal Cancer Screening Rate to 80%? *Clinical gastroenterology and hepatology : the official clinical practice journal of the American Gastroenterological Association*. 2021;19(4):633-645. doi:[10.1016/j.cgh.2019.12.016](https://doi.org/10.1016/j.cgh.2019.12.016) | colorectal | 2021 |
| 1. Yu C, Skootsky S, Grossman M, et al. A Multi-Level Fit-Based Quality Improvement Initiative to Improve Colorectal Cancer Screening in a Managed Care Population. *Clin Transl Gastroenterol*. 2018;9(8):177. doi:[10.1038/s41424-018-0046-z](https://doi.org/10.1038/s41424-018-0046-z) | colorectal | 2018 |
| 1. Yadav S, Hartkop S, Cardenas PY, et al. Utilization of a breast cancer risk assessment tool by internal medicine residents in a primary care clinic: impact of an educational program. *BMC cancer*. 2019;19(1):228. doi:[10.1186/s12885-019-5418-6](https://doi.org/10.1186/s12885-019-5418-6) | breast | 2019 |
| 1. Mazur et al., 2017, Enhancing Providers Ability to Follow-up on Abnormal Test Results. Final Report. Agency for Healthcare Research and Quality. Digital Healthcare Research. | breast, cervical | 2017 |
| 1. Skinner CS, Halm EA, Bishop WP, et al. Impact of Risk Assessment and Tailored versus Nontailored Risk Information on Colorectal Cancer Testing in Primary Care: A Randomized Controlled Trial. *Cancer epidemiology, biomarkers & prevention : a publication of the American Association for Cancer Research, cosponsored by the American Society of Preventive Oncology*. 2015;24(10):1523-1530. doi:[10.1158/1055-9965.EPI-15-0122](https://doi.org/10.1158/1055-9965.EPI-15-0122) | colorectal | 2015 |
| 1. Selby K, Baumgartner C, Levin TR, et al. Interventions to Improve Follow-up of Positive Results on Fecal Blood Tests: A Systematic Review. *Ann Intern Med*. 2017;167(8):565-575. doi:[10.7326/M17-1361](https://doi.org/10.7326/M17-1361) | colorectal | 2017 |
| 1. Tate CE, Matlock DD, Dalton AF, et al. Implementation and Evaluation of a Novel Colorectal Cancer Decision Aid Using a Centralized Delivery Strategy. *Joint Commission journal on quality and patient safety*. 2018;44(6):353-360. doi:[10.1016/j.jcjq.2017.11.009](https://doi.org/10.1016/j.jcjq.2017.11.009) | colorectal | 2018 |
| 1. Thompson B, Carosso EA, Jhingan E, et al. Results of a randomized controlled trial to increase cervical cancer screening among rural Latinas. *Cancer*. 2017;123(4):666-674. doi:[10.1002/cncr.30399](https://doi.org/10.1002/cncr.30399) | cervical | 2017 |
| 1. Lafata et al. *Randomised Trial to Evaluate the Effectiveness and Impact of Offering Postvisit Decision Support and Assistance in Obtaining Physicianrecommended Colorectal Cancer Screening: The e-Assist: Colon Health Study—a Protocol Study*. clinicaltrials.gov; 2020. <https://clinicaltrials.gov/ct2/show/NCT02798224> | colorectal | 2019 |
| 1. Wu CA, Mulder AL, Zai AH, et al. A population management system for improving colorectal cancer screening in a primary care setting. *J Eval Clin Pract*. 2016;22(3):319-328. doi:[10.1111/jep.12427](https://doi.org/10.1111/jep.12427) | colorectal | 2016 |
| 1. Vanderpool RC, Moore SC, Stradtman LR, Carman AL, Kurgat HL, Fain P. Adaptation of an Evidence-Based Intervention to Improve Preventive Care Practices in a Federally Qualified Health Center in Appalachian Kentucky. *J Health Care Poor Underserved*. 2016;27(4A):46-52. doi:[10.1353/hpu.2016.0185](https://doi.org/10.1353/hpu.2016.0185) | breast, colorectal, cervical | 2016 |
| 1. Wilson FA, Villarreal R, Stimpson JP, Pagán JA. Cost-effectiveness analysis of a colonoscopy screening navigator program designed for Hispanic men. *J Cancer Educ*. 2015;30(2):260-267. doi:[10.1007/s13187-014-0718-7](https://doi.org/10.1007/s13187-014-0718-7) | colorectal | 2015 |
| 1. Winer RL, Lin J, Tiro JA, et al. Effect of Mailed Human Papillomavirus Test Kits vs Usual Care Reminders on Cervical Cancer Screening Uptake, Precancer Detection, and Treatment A Randomized Clinical Trial. *JAMA NETWORK OPEN*. 2019;2(11). doi:[10.1001/jamanetworkopen.2019.14729](https://doi.org/10.1001/jamanetworkopen.2019.14729) | cervical | 2019 |
| 1. Woolf SH, Krist AH, Lafata JE, et al. Engaging Patients in Decisions About Cancer Screening: Exploring the Decision Journey Through the Use of a Patient Portal. *Am J Prev Med*. 2018;54(2):237-247. doi:[10.1016/j.amepre.2017.10.027](https://doi.org/10.1016/j.amepre.2017.10.027) | breast, colorectal | 2018 |
| 1. Wang GX, Pizzi BT, Miles RC, et al. Implementation and Utilization of a “Pink Card” Walk-In Screening Mammography Program Integrated With Physician Visits. *Journal of the American College of Radiology : JACR*. 2020;17(12):1602-1608. doi:[10.1016/j.jacr.2020.07.007](https://doi.org/10.1016/j.jacr.2020.07.007) | breast | 2020 |
| 1. Weiner BJ, Rohweder CL, Scott JE, et al. Using Practice Facilitation to Increase Rates of Colorectal Cancer Screening in Community Health Centers, North Carolina, 2012-2013: Feasibility, Facilitators, and Barriers. *PREVENTING CHRONIC DISEASE*. 2017;14. doi:[10.5888/pcd14.160454](https://doi.org/10.5888/pcd14.160454) | colorectal | 2017 |
| 1. Ajeesh S, Luis R. A Comprehensive Electronic Health Record Based Patient Navigation Module Including Technology Driven Colorectal Cancer Outreach and Education. *Journal of cancer education : the official journal of the American Association for Cancer Education*. 2018;33(3):627-633. doi:[10.1007/s13187-017-1184-9](https://doi.org/10.1007/s13187-017-1184-9) | colorectal | 2018 |
| 1. Baker DW, Liss DT, Alperovitz-Bichell K, et al. Colorectal Cancer Screening Rates at Community Health Centers that Use Electronic Health Records: A Cross Sectional Study. *J Health Care Poor Underserved*. 2015;26(2):377-390. doi:[10.1353/hpu.2015.0030](https://doi.org/10.1353/hpu.2015.0030) | colorectal | 2015 |
| 1. Mehta SJ, Khan T, Guerra C, et al. A Randomized Controlled Trial of Opt-in Versus Opt-Out Colorectal Cancer Screening Outreach. *Am J Gastroenterol*. 2018;113(12):1848-1854. doi:10.1038/s41395-018-0151-3 | colorectal | 2018 |
| 1. Bakhai S, Ahluwalia G, Nallapeta N, Mangat A, Reynolds JL. Faecal immunochemical testing implementation to increase colorectal cancer screening in primary care. *BMJ open quality*. 2018;7(4):e000400. doi:[10.1136/bmjoq-2018-000400](https://doi.org/10.1136/bmjoq-2018-000400) | colorectal | 2018 |
| 1. Barajas M, Tangka FKL, Schultz J, et al. Examining the Effectiveness of Provider Incentives to Increase CRC Screening Uptake in Neighborhood Healthcare: A California Federally Qualified Health Center. *Health Promot Pract*. 2020;21(6):898-904. doi:[10.1177/1524839920954166](https://doi.org/10.1177/1524839920954166) | colorectal | 2020 |
| 1. Florea KS, Novosel LM, Schlenk EA. Improvement in colon cancer screening through use of a multilevel intervention: A QI initiative. *JOURNAL OF THE AMERICAN ASSOCIATION OF NURSE PRACTITIONERS*. 2016;28(7):362-369. doi:[10.1002/2327-6924.12320](https://doi.org/10.1002/2327-6924.12320) | colorectal | 2016 |
| 1. Berkowitz SA, Percac-Lima S, Ashburner JM, et al. Building Equity Improvement into Quality Improvement: Reducing Socioeconomic Disparities in Colorectal Cancer Screening as Part of Population Health Management. *J Gen Intern Med*. 2015;30(7):942-949. doi:[10.1007/s11606-015-3227-4](https://doi.org/10.1007/s11606-015-3227-4) | colorectal | 2015 |
| 1. Brenner AT, Hoffman R, McWilliams A, et al. Colorectal Cancer Screening in Vulnerable Patients: Promoting Informed and Shared Decisions. *Am J Prev Med*. 2016;51(4):454-462. doi:[10.1016/j.amepre.2016.03.025](https://doi.org/10.1016/j.amepre.2016.03.025) | colorectal | 2016 |
| 1. Brenner AT, Rhode J, Yang JY, et al. Comparative effectiveness of mailed reminders with and without fecal immunochemical tests for Medicaid beneficiaries at a large county health department: A randomized controlled trial. *Cancer*. 2018;124(16):3346-3354. doi:[10.1002/cncr.31566](https://doi.org/10.1002/cncr.31566) | colorectal | 2018 |
| 1. Champion VL, Christy SM, Rakowski W, et al. A Randomized Trial to Compare a Tailored Web-Based Intervention and Tailored Phone Counseling to Usual Care for Increasing Colorectal Cancer Screening. *Cancer Epidemiol Biomarkers Prev*. 2018;27(12):1433-1441. doi:[10.1158/1055-9965.EPI-18-0180](https://doi.org/10.1158/1055-9965.EPI-18-0180) | colorectal | 2018 |
| 1. Cole AM, Esplin A, Baldwin LM. Adaptation of an Evidence-Based Colorectal Cancer Screening Program Using the Consolidated Framework for Implementation Research. *Prev Chronic Dis*. 2015;12:E213. doi:[10.5888/pcd12.150300](https://doi.org/10.5888/pcd12.150300) | colorectal | 2015 |
| 1. Gupta S, Miller S, Koch M, et al. Financial Incentives for Promoting Colorectal Cancer Screening: A Randomized, Comparative Effectiveness Trial. *Am J Gastroenterol*. 2016;111(11):1630-1636. doi:[10.1038/ajg.2016.286](https://doi.org/10.1038/ajg.2016.286) | colorectal | 2016 |
| 1. Meenan RT, Anderson ML, Chubak J, et al. An Economic Evaluation of Colorectal Cancer Screening in Primary Care Practice. *AMERICAN JOURNAL OF PREVENTIVE MEDICINE*. 2015;48(6):714-721. doi:[10.1016/j.amepre.2014.12.016](https://doi.org/10.1016/j.amepre.2014.12.016) | colorectal | 2015 |
| 1. Goldman SN, Liss DT, Brown T, et al. Comparative Effectiveness of Multifaceted Outreach to Initiate Colorectal Cancer Screening in Community Health Centers: A Randomized Controlled Trial. *J Gen Intern Med*. 2015;30(8):1178-1184. doi:[10.1007/s11606-015-3234-5](https://doi.org/10.1007/s11606-015-3234-5) | colorectal | 2015 |
| 1. Goldstein KM, Fisher DA, Wu RR, et al. An electronic family health history tool to identify and manage patients at increased risk for colorectal cancer: protocol for a randomized controlled trial. *TRIALS*. 2019;20(1). doi:[10.1186/s13063-019-3659-y](https://doi.org/10.1186/s13063-019-3659-y) | colorectal | 2019 |
| 1. Green BB, Anderson ML, Chubak J, Fuller S, Meenan RT, Vernon SW. Impact of continued mailed fecal tests in the patient-centered medical home: Year 3 of the Systems of Support to Increase Colon Cancer Screening and Follow-Up randomized trial. *Cancer*. 2016;122(2):312-321. doi:[10.1002/cncr.29734](https://doi.org/10.1002/cncr.29734) | colorectal | 2016 |
| 1. Gagliardi KS, Coleman S, Intinarelli G, et al. An Automated Telephone Call System Improves the Reach and Cost-effectiveness of Panel Management Outreach for Cancer Screening. *The Journal of ambulatory care management*. 2020;43(2):148-156. doi:[10.1097/JAC.0000000000000322](https://doi.org/10.1097/JAC.0000000000000322) | breast, colorectal, cervical | 2020 |
| 1. Green BB, Anderson ML, Cook AJ, et al. A centralized mailed program with stepped increases of support increases time in compliance with colorectal cancer screening guidelines over 5 years: A randomized trial. *Cancer*. 2017;123(22):4472-4480. doi:[10.1002/cncr.30908](https://doi.org/10.1002/cncr.30908) | colorectal | 2017 |
| 1. Mehta SJ, Jensen CD, Quinn VP, et al. Race/Ethnicity and Adoption of a Population Health Management Approach to Colorectal Cancer Screening in a Community-Based Healthcare System. *J Gen Intern Med*. 2016;31(11):1323-1330. doi:[10.1007/s11606-016-3792-1](https://doi.org/10.1007/s11606-016-3792-1) | colorectal | 2016 |
| 1. Mehta SJ, Feingold J, Vandertuyn M, et al. Active Choice and Financial Incentives to Increase Rates of Screening Colonoscopy-A Randomized Controlled Trial. *Gastroenterology*. 2017;153(5):1227-1229.e2. doi:[10.1053/j.gastro.2017.07.015](https://doi.org/10.1053/j.gastro.2017.07.015) | colorectal | 2017 |
| 1. Militello LG, Diiulio JB, Borders MR, et al. Evaluating a Modular Decision Support Application for Colorectal Cancer Screening. *Appl Clin Inform*. 2017;8(1):162-179. doi:[10.4338/ACI-2016-09-RA-0152](https://doi.org/10.4338/ACI-2016-09-RA-0152) | colorectal | 2017 |
| 1. Siembida EJ, Radhakrishnan A, Nowak SA, Parker AM, Pollack CE. Linking Reminders and Physician Breast Cancer Screening Recommendations: Results From a National Survey. *JCO clinical cancer informatics*. 2017;1:1-10. doi:[10.1200/CCI.17.00090](https://doi.org/10.1200/CCI.17.00090) | breast | 2017 |
| 1. Schwartz AR, Levin FL, O’Neil JA Jr, Braithwaite RS. Pilot of Decision Support to Individualize Colorectal Cancer Screening Recommendations. *AMERICAN JOURNAL OF MANAGED CARE*. 2015;21(7):439+. | colorectal | 2015 |
| 1. Schiff GD, Bearden T, Hunt LS, et al. Primary Care Collaboration to Improve Diagnosis and Screening for Colorectal Cancer. *JOINT COMMISSION JOURNAL ON QUALITY AND PATIENT SAFETY*. 2017;43(7):338-350. doi:[10.1016/j.jcjq.2017.03.004](https://doi.org/10.1016/j.jcjq.2017.03.004) | colorectal | 2017 |
| 1. Schapira MM, Hubbard RA, Seitz HH, et al. The Impact of a Risk-Based Breast Cancer Screening Decision Aid on Initiation of Mammography Among Younger Women: Report of a Randomized Trial. *MDM Policy Pract*. 2019;4(1):2381468318812889. doi:[10.1177/2381468318812889](https://doi.org/10.1177/2381468318812889) | breast | 2019 |
| 1. Meyer AN, Murphy DR, Singh H. Communicating Findings of Delayed Diagnostic Evaluation to Primary Care Providers. *J Am Board Fam Med*. 2016;29(4):469-473. doi:[10.3122/jabfm.2016.04.150363](https://doi.org/10.3122/jabfm.2016.04.150363) | colorectal | 2016 |
| 1. Conn ME, Kennedy-Rea S, Subramanian S, et al. Cost and Effectiveness of Reminders to Promote Colorectal Cancer Screening Uptake in Rural Federally Qualified Health Centers in West Virginia. *Health Promot Pract*. 2020;21(6):891-897. doi:[10.1177/1524839920954164](https://doi.org/10.1177/1524839920954164) | colorectal | 2020 |
| 1. Cole AM, Tu SP, Fernandez ME, Calo WA, Hotz J, Wolver S. Reported Use of Electronic Health Records to Implement Evidence Based Approaches to Colorectal Cancer Screening in Community Health Centers. *JOURNAL OF HEALTH CARE FOR THE POOR AND UNDERSERVED*. 2015;26(4):1235-1245. doi:[10.1353/hpu.2015.0120](https://doi.org/10.1353/hpu.2015.0120) | colorectal | 2015 |
| 1. Chubak J, Garcia MP, Burnett-Hartman AN, et al. Time to Colonoscopy after Positive Fecal Blood Test in Four U.S. Health Care Systems. *Cancer Epidemiol Biomarkers Prev*. 2016;25(2):344-350. doi:[10.1158/1055-9965.EPI-15-0470](https://doi.org/10.1158/1055-9965.EPI-15-0470) | colorectal | 2016 |
| 1. Chaudhry R, MacLaughlin KL, Elayavilli RK, et al. NLP-enabled decision support for cervical cancer screening and surveillance. Agency for Healthcare Research and Quality. Final Report. Published online 2017:20. <https://digital.ahrq.gov/ahrq-funded-projects/nlp-enabled-decision-support-cervical-cancer-screening-and-surveillance/final-report> | cervical | 2017 |
| 1. Castaneda SF, Bharti B, Espinoza-Giacinto RA, et al. Evaluating Two Evidence-Based Intervention Strategies to Promote CRC Screening Among Latino Adults in a Primary Care Setting. *JOURNAL OF RACIAL AND ETHNIC HEALTH DISPARITIES*. 2018;5(3):530-535. doi:[10.1007/s40615-017-0395-4](https://doi.org/10.1007/s40615-017-0395-4) | colorectal | 2018 |
| 1. Chuang E, Pourat N, Chen X, et al. Organizational Factors Associated with Disparities in Cervical and Colorectal Cancer Screening Rates in Community Health Centers. *J Health Care Poor Underserved*. 2019;30(1):161-181. doi:[10.1353/hpu.2019.0014](https://doi.org/10.1353/hpu.2019.0014) | colorectal, cervical | 2019 |
