# Supplementary material for: Implementation of Health IT for Cancer Screening in US Primary Care: Scoping Review
Source: JMIR Cancer. 2024 Apr 30;10:e49002. doi: 10.2196/49002 (PMC11094604; doi:10.2196/49002)
Supplement: Multimedia Appendix 7 [file cancer_v10i1e49002_app7.docx]

Appendix 7. Reporting Status of Health Information Technology (HIT) Effectiveness by Cancer Screening Activities and HIT Functions as Represented in Included Studies

|  | **Colorectal Cancer** | | | **Breast Cancer** | | | **Cervical Cancer** | | |
| --- | --- | --- | --- | --- | --- | --- | --- | --- | --- |
|  | Positive | Mixed | Null | Positive | Mixed | Null | Positive | Mixed | Null |
|  | n=36 | n=9 | n=9 | n=9 | n=1 | n=4 | n=7 | n=1 | n=2 |
| **Panel Management** |  |  |  |  |  |  |  |  |  |
| CDS (POC) | 6 (7) | 2 (2) | 1 (1) | - | - | - | - | - | - |
| CDS (PM/Outreach) | 22 (27) | 2 (2) | 6 (7) | 3 (11) | - | 2 (7) | 1 (5) | 1 (5) | 2 (11) |
| Risk Identification | 1 (1) | 1 (1) | 3 (4) | 1 (4) | - | - | 1 (5) | - | - |
| Patient Decision Aid | - | 1 (1) | - | - | - | - | - | - | - |
| Provider Assessment & Feedback | 1 (1) | 1 (1) | - | - | - | - | - | - | - |
| Tracking Patient Adherence | 4 (5) | - | 3 (4) | 1 (4) | - | 1 (4) | 1 (5) | 1 (5) | 1 (5) |
| Other | 1 (1) | - | - | - | - | - | - | - | - |
| **Point of Care** |  |  |  |  |  |  |  |  |  |
| CDS (POC) | 18 (22) | 5 (6) | 4 (5) | 5 (18) | 1 (4) | 2 (7) | 5 (26) | - | 1 (5) |
| CDS (PM/Outreach) | 5 (6) | 1 (1) | 1 (1) | - | - | - | - | - | - |
| Risk Identification | 1 (1) | - | 2 (2) | - | - | - | 1 (5) | - | - |
| Provider Assessment & Feedback | 4 (5) | 1 (1) | - | - | - | - |  | - | - |
| Tracking Patient Adherence | 2 (2) | - | 1 (1) | 1 (4) | - | - | 1 (5) | - | - |
| **Follow-up (Referral)** |  |  |  |  |  |  |  |  |  |
| CDS (POC) | 5 (6) | - | 1 (1) | 2 (7) | - | - | 1 (5) | - | - |
| CDS (PM/Outreach) | 9 (11) | 2 (2) | 3 (4) | 1 (4) | - | 2 (7) | 1 (5) | 1 (5) | 2 (11) |
| Risk Identification | 1 (1) | - | 2 (2) | - | - | - | - | - | - |
| Provider Assessment & Feedback | 1 (1) | 1 (1) | - | - | - | - | - | - | - |
| Tracking Patient Adherence | 10 (12) | 1 (1) | 3 (3) | 2 (7) | - | 1 (4) | 2 (11) | 1 (5) | 1 (5) |
| **Follow-up (Positive/Abnormal Results)** |  |  |  |  |  |  |  |  |  |
| CDS (POC) | - | - | 1 (1) | - | - | - | 2 (11) | - | - |
| CDS (PM/Outreach) | 2 (2) | - | 1 (1) | - | - | - | 1 (5) | - | - |
| Risk Identification | - | - | 2 (2) | - | - | - | 1 (5) | - | - |
| Tracking Patient Adherence | 3 (4) | - | 2 (2) | - | - | - | - | - | - |
| **Acquire Previous Results** |  |  |  |  |  |  |  |  |  |
| CDS (POC) | 1 (1) | - | 1 (1) | - | - | - | 1 (5) | - | - |
| CDS (PM/Outreach) | 1 (1) | - | 2 (2) | - | - | 1 (4) | - | - | 1 (5) |
| Risk Identification | - | - | 2 (2) | - | - | - | 2 (11) | - | - |
| Tracking Patient Adherence | - | - | 2 (2) | - | - | - | - | - | - |

**Footnotes:** **Data is presented as n (%). Percents were calculated with respect to included studies for each cancer screening type category. Some studies featured more than one HIT source, function, and cancer screening activity. As a result, these categories are not mutually exclusive and will not necessarily sum to 100%. Positive: refers to reported results of HIT effectiveness demonstrated improvements in cancer screening uptake, mixed: refers to reported results of HIT effectiveness varied, null: refers to reported results of HIT effectiveness did not demonstrate significant results (p-value < 0.05). Abbreviations captured in this table are detailed in Appendix 4.**
