# Supplementary material for: Implementation of Health IT for Cancer Screening in US Primary Care: Scoping Review
Source: JMIR Cancer. 2024 Apr 30;10:e49002. doi: 10.2196/49002 (PMC11094604; doi:10.2196/49002)
Supplement: Multimedia Appendix 8 [file cancer_v10i1e49002_app8.docx]

Appendix 8. Reporting of Health Information Technology (HIT) Adoption as Represented in Included Studies

|  | **Colorectal Cancer** | **Breast Cancer** | **Cervical Cancer** |
| --- | --- | --- | --- |
|  | n=83 | n=28 | n=19 |
| **Reporting Status** |  |  |  |
| No | 73 (88) | 19 (68) | 18 (95) |
| Yes | 10 (12) | 9 (32) | 1 (5) |
| **Reported Rate of Adoption** |  |  |  |
| 50% or less | 7 (8) | 6 (21) | 1 (5) |
| More than 50% | 5 (6) | 4 (14) | 1 (5) |

**Footnotes: Data is presented as n (%). Percents were calculated with respect to included studies for each cancer screening type category. Some studies featured more than one HIT source, function, and cancer screening activity. As a result, these categories are not mutually exclusive and will not necessarily sum to 100%.**
