# Supplementary material for: Implementation of Health IT for Cancer Screening in US Primary Care: Scoping Review
Source: JMIR Cancer. 2024 Apr 30;10:e49002. doi: 10.2196/49002 (PMC11094604; doi:10.2196/49002)
Supplement: Multimedia Appendix 9 [file cancer_v10i1e49002_app9.docx]

Appendix 9. Barriers and Facilitators (with examples) of Health Information Technology (HIT) Adoption aligned with Integrated Technology Implementation Model (ITIM)

| **ITIM Constructs** | **Barriers** | **Examples** | **Facilitator** | **Examples** |
| --- | --- | --- | --- | --- |
|  | n=34 |  | n=37 |  |
| Adoption / Adopters | 2 (6)^a^ | - Concerns about inaccuracy/conflicting clinical decision support (CDS) recommendations compared to health system recommendations for cancer screening | 1 (3) | - CDS follows United States Preventive Services Taskforce (USPSTF) recommendations for cancer screening |
| Communication | 6 (18) | - Separate communication structures among clinic staff and administrators created challenges across the organization and within teams. | 5 (14) | - Quality improvement staff monitored colorectal cancer rates and provided this information to clinic staff. - Organization incorporated processes for provider assessment and feedback and team networking. |
| Economic Environment | 5 (15) | - Embedding reminder systems in the electronic health record (EHR) might require up-front costs and human capital. - Increased pressure from payers to contain costs and improve performance on quality measures^b^ | 6 (16) | - Federal funder executed contracts with health systems to provide funding support for implementation and provided technical assistance. - Increased pressure from payers to contain costs and improve performance on quality measures* |
| Facilitators (Boundary Spanner) | - | - | 4 (11) | - Designated staff to provide technical assistance and HIT training support. |
| Implementation | 3 (9) | - Alert fatigue (clinicians and patients) - Information Technology (IT) department not involved early enough in program development to determine EHR optimization (e.g., establish automatic call interface from EHR). | 9 (24) | - Included a pre-implementation phase to determine IT infrastructure needs - Incorporated ongoing training for all staff involved in the intervention |
| Inner Context | 17 (50) | - Limited staff time to utilize the HIT tool - HIT adoption competed with other clinic priorities. | 14 (38) | - Buy-in from health system/clinics to improve processes for cancer screening - Organizational policies and existing IT infrastructure to support HIT adoption |
| Interfacing Systems | 5 (15) | - Lack of integration or documentation of reminder calls in the EHR increased risk of duplication of services. | 2 (5) | - Having high-quality electronic systems that can support tracking patients in real time for cancer screening programs |
| Leadership | 2 (6) | - Leadership turnover or not involved | 2 (5) | - Practice champions located in the clinic to support intervention implementation. |
| Nature of the innovation | 15 (44) | - Inaccurate cancer screening data reported in the HIT - The burden of HIT development and maintenance | 17 (46) | - HIT automation and customization features reduced staff resources and reduced time needed in providing care. |
| Outer Context | 11 (32) | - Working with EHR vendor to activate and update the tool was cumbersome - Difficult to assess screening results conducted outside the clinic in which HIT was being used | 9 (24) | - Medicaid expansion promoted cancer screening as an incentivized metric. - Clinic(s) were designated as Federally Qualified Health Centers which necessitated a greater emphasis on quality improvement. |
| Users (adopters) | 9 (26) | - HIT was only limited to English application only - Patient participants without a computer or smartphone may have limited access to HIT intervention. | 4 (11) | - Decreased attitudinal barriers to cancer screening (improvement in self-efficacy and fatalism cancer screening) |
| Workflow | 9 (26) | - Health plan leaders and primary care providers identified that the lack of integration or documentation of the reminder calls in the EHR created challenges by increasing providers’ chances of duplication of effort or service. | 11 (30) | - To support a patient reminder system, a colorectal cancer patient registry was created and a standardized process for identifying and contacting patients who were not up to date with colorectal cancer screening was established. - Linking decision aid invitations to appointment reminders |

**Footnotes: ^a^Data is presented in this table as n (%). Percents were calculated with respect to the total reported barriers (n=34) and facilitators (n=37). Some studies featured both barriers and facilitators to HIT adoption for cancer screening in primary care. As a result, these categories are not mutually exclusive and will not necessarily sum to 100%. ^b^ITIM construct was identified as both a barrier and facilitator.**
