# Supplementary material for: Implementation of Health IT for Cancer Screening in US Primary Care: Scoping Review
Source: JMIR Cancer. 2024 Apr 30;10:e49002. doi: 10.2196/49002 (PMC11094604; doi:10.2196/49002)
Supplement: Multimedia Appendix 10 [file cancer_v10i1e49002_app10.docx]

Appendix 10. Expert Recommendations for Implementing Change (ERIC) Implementation Strategies Used to Support HIT Adoption (n=24 studies)

| **Reference**  *(See Appendix 6 for full citation)* | **Implementation Strategy** | **Cancer Screening Type** | **Actor** | **Action** | **Target of action** | **Temporality** | **Dose** | **Implementation Outcome** | **Justification** |
| --- | --- | --- | --- | --- | --- | --- | --- | --- | --- |
| 1. Coronado et al. 2018 | Create a learning collaborative | colorectal | researchers | meetings, training clinical teams | increase use of HIT^a^ tools | throughout the implementation | monthly | not reported | integrate intervention into existing workflow |
| 3. Kemper et al. 2018 | Centralize technical assistance | colorectal | health organizations | feedback on process flow, standardize processes | establish centralized screening | not reported | not reported | not reported | establish consistent screening implementation, support tool use |
| 4. Kim et al. 2020 | Identify and prepare champions | colorectal | health organizations | identify champion | increase use of HIT-supported EBIs^b^, generate provider reminders | pre-implementation | not reported | not reported | promote tool use, support tool use |
| 4. Kim et al. 2020 | Audit and provide feedback | colorectal | health system or clinic leadership | feedback on screening rate | increase use of HIT-supported EBIs | throughout the implementation | quarterly | not reported | promote tool use |
| 4. Kim et al. 2020 | Facilitate relay of clinical data to providers | colorectal | non-clinical support staff | facilitate reminder system | increase use of HIT tools, generate provider reminders | pre-visit, post-visit | weekly | not reported | integrate intervention into existing workflow |
| 9. Lara et al. 2018 | Access new funding | colorectal | health organizations | application of funding | increase use of HIT-supported EBIs, implement the HIT-supported EBIs | pre-implementation | not reported | not reported | funding support |
| 9. Lara et al. 2018 | Assess for readiness and identify barriers and facilitators | colorectal | public health staff, non-clinical support staff | feedback on process flow | identify needs and readiness of implementing HIT-supported EBIs | pre-implementation | once | not reported | access readiness |
| 9. Lara et al. 2018 | Audit and provide feedback | colorectal | non-clinical support staff | establish the feedback process, feedback on screening rate | engage stakeholders, implement the HIT-supported EBIs | throughout the implementation | monthly | not reported | promote tool use |
| 9. Lara et al. 2018 | Facilitate relay of clinical data to providers | colorectal | non-clinical support staff | facilitate reminder system | implement the HIT-supported EBIs, generate provider reminders | pre-visit | routine | not reported | integrate intervention into existing workflow |
| 9. Lara et al. 2018 | Facilitation | colorectal | public health staff | establish the feedback process | establish provider feedback | pre-implementation | not reported | not reported | promote tool use |
| 15. Kim et al., 2020 | Audit and provide feedback | colorectal | researchers | feedback on screening rate | establish provider feedback, increase use of HIT tools | throughout the implementation | quarterly | not reported | promote tool use |
| 15. Kim et al., 2020 | Conduct educational meetings | colorectal | researchers | training clinical teams | increase cancer screening, improve the implementation of HIT-supported EBIs | throughout the implementation | routine | not reported | promote tool use |
| 18. Levy et al. 2015 | Conduct educational meetings | breast; colorectal; cervical | clinicians, researchers | training clinical teams | increase use of HIT tools | not reported | not reported | not reported | support tool use |
| 19. Liles et al. 2015 | Alter incentive/  allowance structures | colorectal | health organizations | implementation of financial incentives | increase cancer screening, increase use of HIT-supported EBIs | not reported | not reported | not reported | promote tool use |
| 19. Liles et al. 2015 | Make training dynamic | colorectal | researchers | training clinical teams | improve provider awareness, improve the implementation of HIT-supported EBIs | not reported | not reported | not reported | promote tool use |
| 19. Liles et al. 2015 | Conduct educational outreach visits | colorectal | researchers | training clinical teams | improve provider awareness, improve the implementation of HIT-supported EBIs | not reported | not reported | not reported | promote tool use |
| 20. Kruse-Diehr et al., 2021 | Conduct educational outreach visits | colorectal | researchers | training clinical teams | increase follow up, improve the implementation of HIT-supported EBIs | pre-implementation and during-implementation | twice | improved screening knowledge | promote tool use |
| 20. Kruse-Diehr et al., 2021 | Use advisory boards and workgroups | colorectal | public health staff, non-clinical support staff, clinic champion, community leaders | recruitment of community advisory board members | engage stakeholders; improve the implementation of HIT-supported EBIs | during-implementation | four times | not reported | promote tool use |
| 20. Kruse-Diehr et al., 2021 | Alter incentive/allowance structures | colorectal | health organizations | goal setting, implementation of financial incentives | increase capacity to implement HIT-supported EBIs | not reported | not reported | not reported | Promote tool use |
| 21. Luckmann et al. 2017 | Conduct educational meetings | breast | NA | training clinical teams, goal setting | increase use of HIT tools | not reported | 65hr | not reported | support tool use |
| 21. Luckmann et al. 2017 | Conduct ongoing training | breast | researchers | training clinical teams, meetings, training clinical teams | support the implementation of HIT-supported EBIs | throughout the implementation | monthly | not reported | support tool use |
| 25. Modica et al. 2019 | Create a learning collaborative | colorectal | health organizations | training clinical teams; webinars, meetings, telephonic technical assistance | increase cancer screening, support the implementation of HIT-supported EBIs, increase use of HIT-supported EBIs | throughout the implementation | not reported | not reported | promote tool use |
| 37. Joseph et al. 2016 | Access new funding | colorectal | health organizations | application of funding | implement the HIT-supported EBIs, increase follow-up, increase use of HIT-supported EBIs | pre-implementation | not reported | improve adoption of HIT | promote tool use |
| 37. Joseph et al. 2016 | Facilitate relay of clinical data to providers | colorectal | non-clinical support staff, clinical support staff | facilitate reminder system | support the implementation of HIT tools | throughout the implementation | daily | improve adoption of HIT | promote tool use |
| 38. Elliott et al. 2021 | Make training dynamic, Conduct educational meetings, Distribute educational materials | breast; colorectal; cervical | researchers | meetings, training clinical teams | increase use of EHR tools | throughout the implementation | not reported | not reported | support tool use |
| 38. Elliott et al. 2021 | Audit and provide feedback | breast; colorectal; cervical | researchers | feedback on process flow | establish provider feedback | throughout the implementation | not reported | improve adoption of HIT | promote tool use |
| 38. Elliott et al. 2021 | Assess for readiness and identify barriers and facilitators | breast; colorectal; cervical | researchers | meetings | identify influences of implementation | throughout the implementation | not reported | not reported | promote tool use |
| 38. Elliott et al. 2021 | Stage implementation scale up | breast; colorectal; cervical | researchers | pilot test the implementation of HIT-supported EBIs | test feasibility of using EHR tools | during-implementation | not reported | not reported | integrate intervention into existing workflow |
| 38. Elliott et al. 2021 | Use advisory boards and workgroups | breast; colorectal; cervical | researchers, patients | feedback on process flow | engage stakeholders; improve the implementation of HIT-supported EBIs | during-implementation | not reported | not reported | engage stakeholders, support tool development and implementation |
| 39. Bowen et al. 2018 | Make training dynamic | breast; colorectal; cervical | researchers | training clinical teams | increase use of HIT tools | not reported | not reported | not reported | support tool use |
| 55. Yu et al 2018 | Remind clinicians | colorectal | researchers | reminders for clinicians | increase use of HIT tools | throughout the implementation | routine | not reported | promote tool use |
| 56. Yadav et al 2019 | Conduct educational meetings, Distribute educational materials | breast | researchers | training clinical teams | increase cancer screening, increase use of HIT tools | not reported | once | increase knowledge of using HIT-tools, improve adoption of HIT-tools | promote tool use |
| 56. Yadav et al 2019 | Provide local technical assistance, Change physical structure and equipment | breast | non-clinical support staff | EHR shortcut | increase use of HIT tools | point of care | routine | improve adoption of HIT | support tool use |
| 63. Wu et al. 2016 | Provide clinical supervision & Use train-the-trainer strategies | colorectal | clinicians | training clinical teams | increase cancer screening, support the implementation of HIT-supported EBIs | pre-implementation | not reported | not reported | support tool use |
| 63. Wu et al. 2016 | Conduct local consensus discussion | colorectal | researchers | feedback on process flow, engage stakeholders, meetings | increase cancer screening, improve the implementation of HIT-supported EBIs | throughout the implementation | routine | not reported | integrate intervention into existing workflow |
| 63. Wu et al. 2016 | Facilitation | colorectal | researchers | facilitate implementation of HIT-supported EBIs | increase use of HIT-supported EBIs | throughout the implementation | routine | not reported | support tool use, promote tool use |
| 63. Wu et al. 2016 | Stage implementation scale up | colorectal | researchers | stepwise implementation in study sites | establish weekly system improvement | throughout the implementation | once | not reported | integrate intervention into existing workflow |
| 64. Vanderpool et al. 2016 | Access for readiness and identify barriers and facilitators | breast; colorectal; cervical | researchers | focus groups, patient interview | identify facilitators and barriers to implementing HIT-supported EBIs, adapt the HIT-supported EBIs | pre-implementation | once | not reported | integrate intervention into existing workflow |
| 64. Vanderpool et al. 2016 | Develop academic partnership | breast; colorectal; cervical | academic institutes, health organizations | forming an academic community partnership | increase use of HIT-supported EBIs | pre-implementation | not reported | not reported | promote tool use |
| 64. Vanderpool et al. 2016 | Stage implementation scale up | breast; colorectal; cervical | NA | feedback on process flow | adapt the HIT-supported EBIs | throughout the implementation | not reported | not reported | integrate intervention into existing workflow |
| 69. Weiner BJ et al. 2017 | Facilitation | colorectal | non-clinical support staff | site visits, configure office systems | support the implementation of HIT-supported EBIs | during-implementation | routine | not reported | integrate intervention into existing workflow |
| 73. Bakhai et al. 2018 | Conduct cyclical small tests of change | colorectal | clinicians, non-clinical support staff, clinical support staff, HIT technicians, public organization staff | feedback on process flow, develop new process flow | increase cancer screening, improve the implementation of HIT-supported EBIs | throughout the implementation | routine | not reported | integrate intervention into existing workflow |
| 75. Florea, et al. 2016 | Conduct educational meetings | colorectal | researcher or quality improvement NP | presentations on the project | improve the implementation of HIT-supported EBIs | pre-implementation | not reported | not reported | promote tool use |
| 80. Cole et al. 2015 | Build a coalition, Promote adaptability | colorectal | researchers | standardize processes | support the implementation of HIT-supported EBIs | pre-implementation | not reported | not reported | integrate intervention into existing workflow |
| 84. Goldstein et al. 2019 | Conduct educational meetings | colorectal | researchers | training clinical teams | support the implementation of HIT-supported EBIs | during-implementation | not reported | not reported | support tool use |
| 93. Schiff et al. 2017 | Conduct cyclical small tests of change | colorectal | health organizations | NA | NA | during-implementation | routine | not reported | integrate intervention into existing workflow |
| 93. Schiff et al. 2017 | Create a learning collaborative | colorectal | clinic leadership | clarifying roles, improving referrals | NA | during-implementation | routine | not reported | promote tool use |
| 93. Schiff et al. 2017 | Facilitation | colorectal | non-clinical support staff | training clinical teams | NA | during-implementation | as needed | not reported | support tool use |
| 93. Schiff et al. 2017 | Involve patients/consumers and family members | colorectal | patients | engage stakeholders, feedback on process flow | NA | during-implementation | not reported | not reported | integrate intervention into existing workflow |
| 96. Conn et al. 2020 | Facilitation | colorectal | researchers | NA | NA | during-implementation | not reported | not reported | integrate intervention into existing workflow, support tool use |
| 96. Conn et al. 2020 | Conduct educational meetings | colorectal | researchers | NA | NA | not reported | not reported | not reported | support tool use |

^a^HIT; health information technology

^b^EBIs; evidence-based interventions
